# A post-fire reforestation assessment and prioritization tool for the Western United States

**DOI:** 10.1186/s42408-025-00405-z

**Published:** 2025-12-11

**Authors:** Zachary A. Holden, Ellen Jungck, Kimberley T. Davis, Dyer A. Warren, Alan Swanson, Solomon Z. Dobrowski, Marco Maneta, Kyle C. Rodman, Lewis Faller, Vince Archer

**Affiliations:** 1 USDA Forest Service, Rocky Mountain Research Station, Missoula, MT 59808 USA; 2 USDA Forest Service Region 1, Missoula, MT 59808 USA; 3 Department of Community and Public Health, University of Montana, Missoula, MT 59810 USA; 4 Department of Forest Management, University of Montana, Missoula, MT 59810 USA; 5 Department of Geosciences, University of Montana, Missoula, MT 59810 USA; 6 Ecological Restoration Institute, Northern Arizona University, Flagstaff, AZ 86011 USA

## Abstract

**Background:**

Increasing wildfire area burned has left millions of hectares in the western United States (US) in need of reforestation. Recent federal legislation allows for increased investments in tree planting to address the backlog of planting needs in previously burned areas. To support post-fire planning and assessment, we developed Regenmapper, a web-based decision support system (DSS) that provides spatial information on natural regeneration potential within post-fire environments. The program is freely available from a web browser (https://alpheus.dbs.umt.edu/regenmapper) and is designed to function across all land ownership categories for the 11 western States.

**Results:**

Regenmapper allows users to select historical wildfires or upload their own burn severity maps for recent fires. Within the burned area, it then predicts the potential for natural regeneration based on distance to mature live trees (seed sources) and hydroclimatic conditions. To this end, we developed 30-m resolution soil water balance and surface temperature models with corresponding projections for the 2050 period based on scenarios from the 6th Coupled Model Intercomparison Project (CMIP6). These data are used to estimate the probability of natural seedling regeneration based on historical or future biophysical conditions, respectively, and species-specific climatic tolerances. We also implement a simple planting prioritization algorithm based on distance to roads and the relative effects of dispersal and climatic limitations to rapidly identify accessible sites that are unlikely to reforest naturally. For US Forest Service managers, we develop an additional prioritization matrix based on fire severity, the probability of natural regeneration, and where federal law mandates reforestation when fires burn through recently harvested areas. Finally, we demonstrate model outputs in a case study approach through the 2017 Lolo Peak fire in Montana, US.

**Conclusions:**

Investments in tree planting will influence the extent and trajectory of future forests, but drought, climate change, and wildfires may challenge the ability of managers to re-establish forests over upcoming decades. DSS’s like Regenmapper will benefit the planning and execution of tree planting efforts by reducing time required to conduct post-fire assessments and improving planting outcomes.

**Supplementary Information:**

The online version contains supplementary material available at 10.1186/s42408-025-00405-z.

## Resumen

**Antecedentes:**

El incremento del área quemada ha dejado millones de hectáreas en el oeste de los EEUU con la necesidad de ser reforestadas. La reciente legislación federal permite las inversiones en plantación de árboles para cubrir la necesidad de alcanzar el trabajo pendiente de reforestar áreas previamente quemadas. Para apoyar el planeamiento y determinación de acciones en el post-fuego, desarrollamos el “*Regenmapper*”, un sistema de soporte de decisiones basado en la web (DDS), que provee de información espacial sobre el potencial de la regeneración natural dentro de ambientes post-incendios. El programa es provisto de manera gratuita desde una página WEB (https://alpheus.dbs.umt.edu/regenmapper) y fue diseñado para que funcione en todos los tipos de categorías de tenencia de la tierra para los 11 estados del oeste de los EEUU.

**Resultados:**

El “*Regenmapper*” permite a los usuarios seleccionar los incendios históricos pasados e ir cargando sus propios mapas de severidad de fuegos recientes. Dentro del área quemada, el programa predice luego el potencial para la regeneración natural basado en la distancia a los árboles maduros (fuentes de semilla) y las condiciones hidro-climáticas. Posteriormente, desarrollamos modelos de balance agua en el suelo y de temperaturas del suelo superficial con sus correspondientes proyecciones para el período 2050, basados en escenarios del 6to. Proyecto Acoplado de Entre-Comparaciones (CMIP6). Estos datos son usados para estimar la probabilidad de la regeneración natural de semilla basada en datos históricos o proyectados a futuro de condiciones biofísicas, respectivamente, y de tolerancias especie-específicas. Implementamos también un algoritmo de priorización de plantación basado en la distancia a rutas o calles y los efectos relativos de la dispersión y las limitaciones climáticas para identificar rápidamente sitios accesibles que sean no factibles de ser reforestados naturalmente. Para los gestores del Servicio Forestal, desarrollamos una matriz de priorizaciones basada en la severidad del fuego, la probabilidad de regeneración natural, y donde la ley federal exige la reforestación, cuando los incendios ocurrieran en áreas recientemente cosechadas. Finalmente, demostramos los resultados de la aplicación del modelo a un estudio de caso del incendio ocurrido en 2017 en *Lolo Peak*, en Montana, EEUU.

**Conclusiones:**

Las inversiones en la plantación de árboles va a tener influencia en la extensión y trayectoria de futuros bosques, aunque la sequía, el cambio climático y lo incendios pueden desafiar la habilidad de los gestores para re-establecer los bosques en décadas por venir. Tanto el DDS´s como el *Regenmapper* beneficiarán el planeamiento y la ejecución de los esfuerzos de plantación mediante la reducción del tiempo requerido para realizar las determinaciones post-fuego y mejorar los resultados de la plantación.

## Background

In recent decades, resource managers in the western United States (US) have seen a dramatic increase in reforestation needs driven largely by increased wildfire burned area (Dumroese et al. 2019; Dobrowski et al. 2024). Consequently, the demand for tree planting activities now outpaces the ability of managers to complete them. The initial post-disturbance reorganization phase, when most planting projects are planned and executed, is a critical indicator of ecosystem resilience to disturbance and has lasting implications for the future of forested ecosystems (Seidl and Turner 2022). Indeed, the importance of understanding and predicting recruitment dynamics in the western US has become evident as much of the region has become more arid, with more frequent droughts and high temperatures that have been linked to widespread forest mortality (Moss et al. 2024) and recruitment failure (Stevens-Rumann et al. 2018; Davis et al. 2019, 2023).

Recent legislation, including passage of the Repairing Public Lands by Adding Necessary Trees (REPLANT) Act within the 2021 Bipartisan Infrastructure Investment and Jobs Act (IIJA), allow for additional investments in tree planting, primarily to address a backlog of reforestation needs in areas burned by wildfires. Given the massive land base that could potentially experience planting (Cook-Patton et al. 2020), meeting that need requires ramping up production at all levels of the supply chain, including seed collection, seedling production, storage, transportation, and field delivery (Fargione et al. 2021). Moreover, labor and housing markets limit hiring ability, which could further constrain capacity (Altieri et al. 2023). These factors, and unforeseen forest losses in the next decade could, despite best efforts, prevent forest managers from meeting objectives set out in REPLANT and other policy directives. Careful selection of sites, with planting designed to maximize survival, will be important for maximizing forest recovery and return on investment. While regional datasets and tools have been developed to assist reforestation efforts using particular tree species in California (Stewart et al. 2021) and the southern Rocky Mountains (Rodman et al. 2022), tools for broad-scale, consistent mapping across the West are not yet available.

Post-fire tree regeneration of many western US conifers is constrained by both the availability of tree seeds and post-fire climatic conditions (Hansen et al. 2018, Davis et al. 2019, Stevens-Rumann and Morgan 2019). Seed availability acts as an initial filter on tree regeneration, but climate can also limit new seedling establishment, even when seeds are available from nearby trees (Davis et al. 2019, Rodman et al. 2020a, b; Holden et al. 2024). Indeed, while tree planting can help to bypass initial filters of seed availability and germination, hydroclimate is strongly tied to regeneration success, with reduced survival of planted trees in warm, dry areas like the Southwest (Ouzts et al. 2015; Rodman et al. 2024), and on arid sites within the southern Rocky Mountains (Marshall, et al. 2024). Identifying locations where natural tree regeneration is likely to occur after fire, as well as key factors limiting tree regeneration (i.e., climate vs. seed availability) will provide valuable information for initial post-fire planning and prioritization of reforestation activities.

Here, we describe Regenmapper—a new, web-based reforestation decision support system (DSS) designed to help managers meet the growing need for post-fire planting information across the western United States. Specifically, we (1) develop models to predict post-fire tree recruitment using high spatial resolution biophysical layers and a large (ca. 10,000 field plots) database of post-fire observations, (2) integrate these models into a publicly available web-based tool for evaluating regeneration potential for historical or recent fires, (3) develop logic for prioritizing planting for both meeting federal law and on biophysical and cost considerations, and (4) return raster- and vector-based spatial data, as well as visual aids to inform post-fire planning.

## Methods

**Fig. 1:**
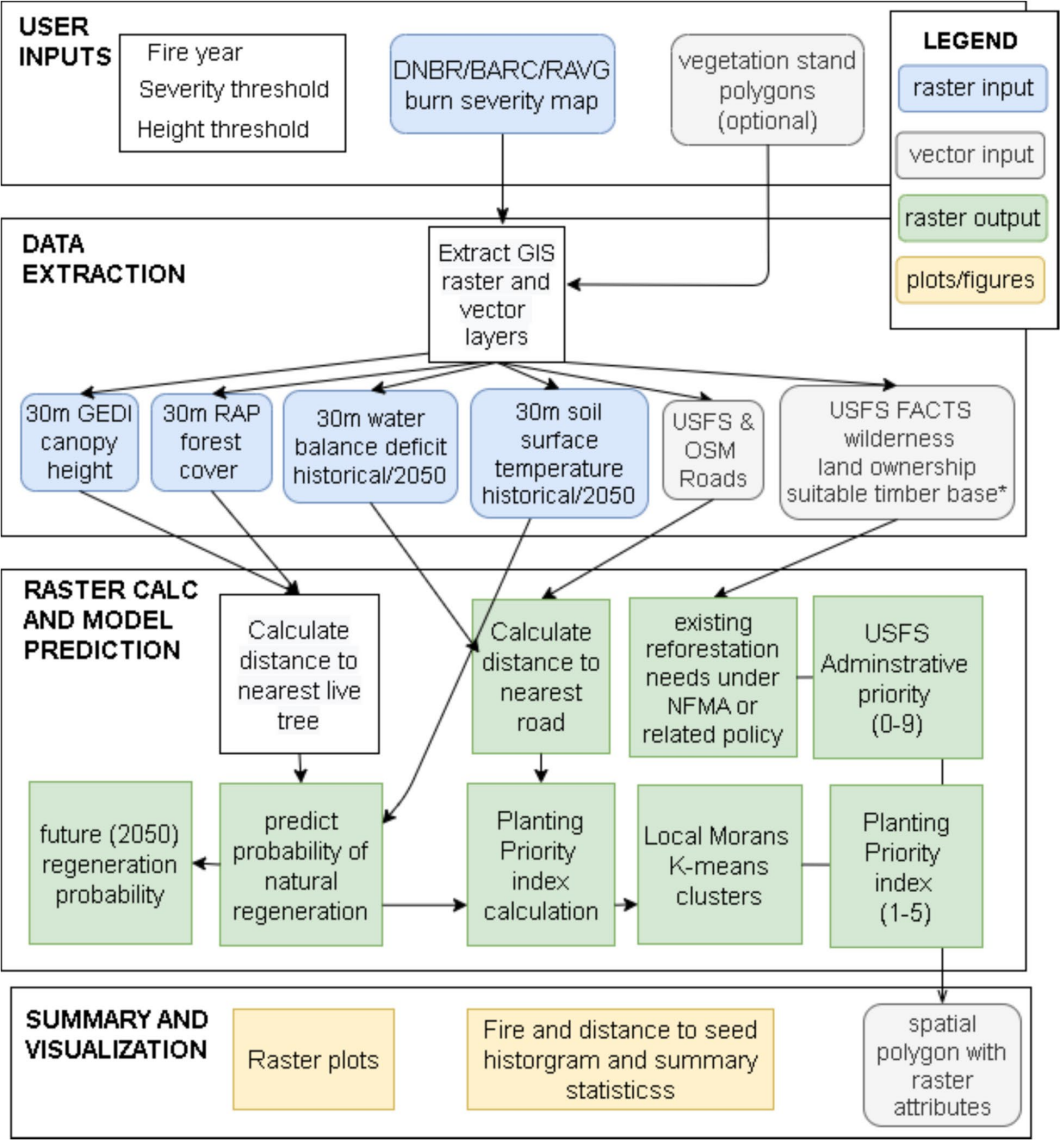
A diagram illustrating the basic Regenmapper workflow and outputs for assessing potential for natural regeneration in post-fire environments

The primary function of Regenmapper is to provide managers across a range of landownership categories with easily accessible data about the post-fire environment at recently disturbed sites. Key outputs include 30-m resolution maps estimating the probability of tree regeneration for common western conifer species, and layers describing limitations related to climate and seed availability. A diagram illustrating the modeling process is shown in Fig. 1.

### Spatial predictors of post-fire recruitment

#### Historical and future hydroclimatic data

We represent the long-term biophysical and climatic conditions at each site using 30-m resolution 95th percentile annual maximum potential soil surface temperature (PSST) grids (Holden et al. 2024) and a 30-m climatic water balance deficit (CWD) grid. To supplement pre-existing maps of PSST, we developed the CWD layer for the 1992–2021 normal period using time series of daily weather data generated at a dense network of points. These data were previously used to map PSST and additional details on the point sampling distribution and mapping methods can be found in Holden et al. (2024). At each of 61,000 sample points, we simulate the daily water balance from 1992 to 2021 with a snow and soil moisture model, using weather inputs (temperature, humidity, radiation, wind speed) extracted from 250-m-resolution daily grids (Holden et al. 2018). We then use data from the 6th Coupled Model Intercomparison Project (CMIP6; Eyring et al. 2016) and a stochastic weather simulator (Steinscheider and Brown 2013) to generate daily weather inputs for the 2050 mean period (2035–2065) at each sample point, using monthly mean deviations in temperature and precipitation based on five general circulation models (GCMs; Table S1). For each variable of interest (e.g., CWD), we calculate the climatological average value (e.g., 1992–2021 or 2035–2065 mean annual climatic water deficit) for each point and then interpolate those values using geographically weighted regression with elevation and solar radiation as covariates. This point-based modeling approach allows for gridding the water balance outputs at a relatively fine 30-m spatial resolution, which would be computationally intractable when applying the same algorithms to high-resolution grids. Additional details on the gridding process are provided in the supplementary materials.

#### Post-fire seed availability

Access to nearby seeds from live trees is a primary constraint on post-fire conifer regeneration (Stevens-Rumann & Morgan 2019; Davis et al. 2023). We developed two alternative methods for estimating pre-fire vegetation cover and distance to live trees. For recent (2021–present) wildfires, pre-fire forest canopy cover is extracted from the Rangeland Analysis Platform (RAP) v.3.0 dataset (Allred et al. 2021), which provides annual 30-m raster grids for 6 vegetation cover types (i.e., shrub, grass, tree) across the continental US. For an enlarged area around the fire extent, we classify any tree cover values > 10% as pre-existing forest. We then overlay these maps with Landsat-derived burn severity maps to calculate the distance to the nearest unburned or low-severity pixel that was forest prior to the fire. This is the primary distance to seed source input used in the model. As an alternative method for earlier (pre-2021) fires, canopy height data from the fusion of spaceborne LiDAR (i.e., GEDI) and Landsat data are also available (Potapov et al. 2021). Here, users have the option to select a height threshold above which surrounding vegetation is considered to be a seed-bearing tree. Figure S1 shows examples of distance to seed source estimates using both GEDI and RAP vegetation models.

### Post-fire recruitment models

We used data on post-fire tree seedling recruitment assembled by Davis et al. (2023) to develop binomial (logit link) generalized linear mixed models (GLMMs) of post-fire recruitment potential. While our methods follow those described by Davis et al. (2023), several hundred plots were omitted at the request of individual data contributors or because they were beyond the geographic scope of this tool (i.e., South Dakota), thus our results may vary slightly from those described in the previous study. The reduced data included 10,100 plots in 322 unique fires across the western US (Figure S10).

Prior to fitting GLMMs, we used boosted regression tree models (BRT; Friedman 2001, Friedman 2002) to search for potential two-way interaction terms. Variable plot sizes in these data require the use of an offset term, precluding the use of common machine learning algorithms like BRT for final predictions. Here, we used BRTs as an exploratory tool, treating log (plot area) as a predictor in the model. We then used spatial cross-validation and feature selection, repeated for a range of different random spatial data configurations, to identify important predictors. For these data, we randomly assigned observations into groups by fire ID using a range of *k* = 3–20 CV folds (approx. 33–5% of the data withheld in each fold). For each of the 14 iterations of the data, we optimized a BRT model using forward feature selection (Meyer et al. 2018), retaining selected variables each time and then summarized the occurrence of individual predictor variables across all 14 iterations (Erickson et al. 2023). This approach provides a robust way of identifying an optimal set of predictor variables for a given dataset. Using a reduced variable model, we then fit a final BRT model for each of the 14 CV scenarios and queried the model for significant 2-way interactions which we then evaluated as candidate predictors in GLMM models. BRT analysis showed some support for clay and sand fractions and interactions with CWD, and a strong interaction between CWD and length of time between a fire and when field sampling occurred.

We developed GLMMs for seven individual conifer species which are widely distributed in the US West (subalpine fir, *Abies lasiocarpa*; western larch, *Larix occidentalis*; Engelmann spruce, *Picea engelmannii*; lodgepole pine, *Pinus contorta*; Douglas-fir: *Pseudotsuga menziesii*; and a combined model for Jeffrey pine, *P. jeffreyi* and ponderosa pine, *P. ponderosa*). Additionally, we developed an all-species model, where any juvenile conifer density greater than zero was coded as a positive response, which we provide for users conducting rapid post-fire assessments where the primary concern is identifying areas where any regeneration is unlikely. We modified individual and all-species species models from Davis et al. (2023) by replacing the 30-year mean climate variables and the heat load index with the finer resolution CWD and PSST metrics described above and then repeated model selection with the new climate variables for all species. All candidate models included time since fire when plots were sampled (years), distance to seed source (nearest adult live tree), percent surrounding post-fire live tree cover (in a 300-m radius), 30-year mean annual CWD, and PSST as fixed effects. The initial full model for each species also included maximum and minimum growing season (April–September) CWD anomaly in the 5 years post-fire, percent soil clay content (Ramcharan, et al. 2018) and its interaction with 30-year CWD, the interaction between time since fire and 30-year CWD, the interaction between PSST and fire severity, and interactions between post-fire CWD anomalies and the following variables: 30-year CWD, PSST, and either fire severity or surrounding tree cover. All candidate models also included fire ID as a random intercept term and an offset of log(plot size). Models were too complex if we included interactions between post-fire CWD anomalies and both fire severity and surrounding tree cover, so we selected one by comparing AIC of initial models. We then used tenfold cross validation to iteratively remove interaction terms, post-fire climate anomaly variables, and soil clay content to maximize model skill based on cross-validated AUC. If removing an interaction or variable resulted in higher cross-validated model skill, then we removed the interaction/variable that resulted in the greatest increase and repeated the cross-validation until removing interactions/variables reduced model skill. Where cross-validated AUC values were within 0.005 of each other, we chose the model with the lowest AIC. Plots located within the same fire were all included within a single fold and predictions to fires used for validation assumed the fire-level intercept was equal to the global intercept.

We report both the full (i.e., no independently withheld data) and cross-validated model accuracy. We used partial effects plots to interpret fixed effects in the final GLMMs, where the effect of the target variable was evaluated across the full range of the data with all other predictors set to their median value. Model predictions are made using values of 100 m^2^ for plot area and 5 years for time since fire; thus, regeneration potential is defined as one more seedlings being present at 5 years post-fire in a 100-m^2^ area. Finally, predictions from the final GLMM models were classified using a threshold calculated by maximizing the sum of the model sensitivity and specificity (Freeman and Moisen 2008).

### Planting prioritization

#### Cost and biophysical conditions

Following large 5 years, it may not be possible to plant in each location that there is an identified reforestation need. To this end, we developed a planting prioritization index (PPI) which balances access considerations, potential for natural tree regeneration, and climatic conditions that may influence planting outcomes.

The index is calculated using the following equation:

$${Planting\;Priority\;Index}_i=\left(\frac1{RoadDistance)}\right)\ast RegenClim\ast(1-RegenProb)$$

where *RoadDistance* (a simple proxy for planting cost) is the distance from the center of each 30-m pixel to the nearest road extracted from OpenStreetMap (OSM) and USFS road databases, *RegenClim* is the probability of natural regeneration, assuming that a seed source is available (climate constraints only), and *RegenProb* is the predicted probability of natural regeneration considering all effects (climate and distance to seed). Here, we isolate climatic effects from the regression model by setting distance to adult trees to a constant value of 10 m and surrounding tree cover to the median dataset value (17%) for all raster cells prior to prediction. The PPI function is applied to each 30-m grid cell within the fire and assigns higher values to cells that are near roads, have a low probability of regenerating naturally, and a high climatic suitability. Figure S11 shows the relative effects of each term across a range of possible values. To further simplify the resulting 30-m resolution cost index raster, we classify the output into 5 discrete classes within each fire following Holden and Evans (2010). First, we calculate local Moran’s *I* statistic on the cost index raster to identify groups of adjacent pixels with similar values. Then we apply K-means clustering to group the Moran’s *I* values into discrete classes. The result is a set of spatially discrete units, ranked from low to high (1–5) with the largest values indicating sites near roads that are unlikely to regenerate without planting and where climatic conditions make successful planting more likely.

#### Administrative prioritization

For US Forest Service managers, factors such as suitability for timber production and land designations (e.g., wilderness areas) help determine management options. Some prior management activities (e.g., regeneration harvest or final harvest) are required to be reforested within 5 years under the National Forest Management Act (NFMA) of 1976. These administrative factors do not change when a site burns and influence management prioritization after a wildfire. Here, we developed a decision matrix to guide planting decisions, based primarily on these policy considerations and our projections of potential for natural regeneration (Table 1). We use a 10-class (0–9) scale that prioritizes pre-existing planting needs and severely burned areas within FS lands managed for timber production. Using the spatial extent of the fire boundary, we query the Forest Activities Tracking System (FACTS), a database of management activities on USFS lands, retaining any spatial polygons with pre-existing reforestation needs (Table S10). Then, using national wilderness boundaries, special land designations, and regional suitable timber base layers (where available), we apply logic from the decision matrix (Table 1) to 30-m fire severity and land ownership raster layers to produce spatial maps of administrative priorities. Currently, these data are integrated in Regenmapper for US Forest Service Regions 1, 2, and 3, and we plan to assemble these datasets for the remaining western regions in the near-future.

**Table 1:** Decision matrix for post-fire planting prioritization on U.S. Forest Service land in the intermountain west. This prioritization integrates policy considerations that apply to U.S. Forest Service managers (see methods) and projections of potential for natural regeneration. Pre-existing needs are determined using spatial data from FACTS and relevant activity codes are listed in supplementary table S10

Fire severity	Pre-existing need	Suitable base	Natural regen predicted	*Regen mapper* cell value	Priority	Diagnoses	Potential FACTS activity
High or Moderate	Yes	N/A	N	9	Very High	Plant	Plan planting in year 3 post disturbance; plan pre-treatment exam for reforestation year 1
High or Moderate	Yes	N/A	Y	8	Very High	Natural Regen	Initiate Natural Regeneration (NRG) and plan certification of natural regeneration without site preparation
High or Moderate	No	Yes	N	7	High	Plant	See *Regenmapper* Cell Value = 9 Potential FACTS Activity Sequence
High or Moderate	No	Yes	Y	6	High	Natural Regen	Initiate NRG and plan certification of natural regeneration without site preparation; plan pretreatment exam for reforestation year 1
High or Moderate	No	No	N	5	Medium	Plant	See *Regenmapper* Cell Value = 9 Potential FACTS Activity Seq
High or Moderate	No	No	Y	4	Medium	Natural Regen	See *Regenmapper* Cell Value = 6 Potential FACTS Activity Seq
Any	N/A	No or N/A	Y or N	3	Variable	Natural Recovery, OR	Natural Recovery; plan follow-up monitoring in 20 years; OR
						Natural Regen. or Plant depending on objectives	Plan certification of natural regen. without site preparation or planting depending on management objectives. See respective *Regenmapper* Cell Values = 6–7 for Potential FACTS Activity Seq
Low	Yes	N/A	Y	2	Medium	Natural Regen	Initiate NRG and plan certification of natural regeneration without site preparation Plan pretreatment exam for reforestation year 1 and stocking survey year 3
Low	No	Yes	Y	1	N/A	No Refo. Action/Stand Improvement (SI) Need, OR	Site may be adequately stocked; assess and plan SI activities stocked. If not adequately stocked, consider natural regeneration, OR
			Y	1	Low	Natural Regen	See *Regenmapper* Cell Value = 2 Potential FACTS Activity Sequence
Any	N/A	No	N/A	0	N/A	Natural Recovery	Natural Recovery; plan follow-up monitoring in 20 years

## Results

### Post-fire conifer regeneration models

**Fig. 2:**
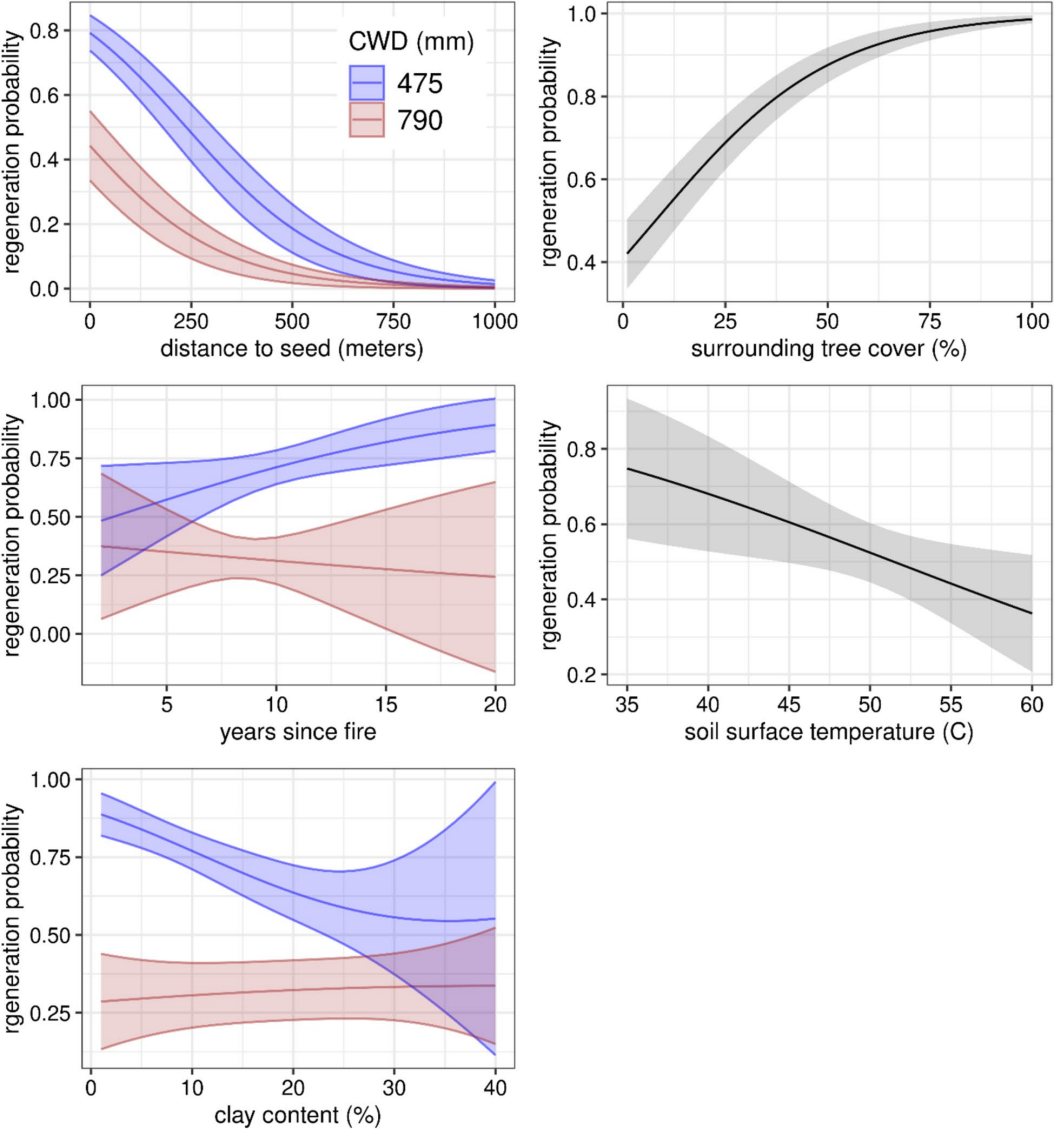
Marginal effects plots for the GLMM model predicting post-fire recruitment for all species combined. Shaded bands represent 95% confidence intervals

Here, we illustrate updated GLMMs that are used to predict the probability of tree recruitment within Regenmapper. The selected “all species” model predicted the probability of recruitment with a cross-validated AUC of 0.74 (80% correctly classified). Coefficients and standard errors are shown in Table 1 and marginal effects plots for model predictors are shown in Fig. 2. Regeneration probability declined with increasing distance to seed source, lower surrounding tree cover, and higher soil surface temperatures. Clay content and its interaction with CWD suggest that high soil clay content in wet environments negatively affects post-fire regeneration, while in more arid sites, it may have a neutral or slight buffering effect against moisture extremes, presumably by increasing moisture retention. The interaction between CWD and time since fire similarly shows contrasting effects for wet and dry areas and implies that the years between the fire and sampling date acts something like an exposure term, with wet areas showing increased probability of recruitment over time, and dry areas showing the opposite pattern. Results for the six individual species models, including marginal effects plots, are shown in Figs. S3-S9 and Tables S3-8. The accuracy of these models ranges from AUC = 0.67 for Engelmann spruce to 0.77 for subalpine fir. Summaries for all models including full and cross-validated accuracies are shown in table S9.

### 2017 Lolo Peak fire case study

**Fig. 3:**
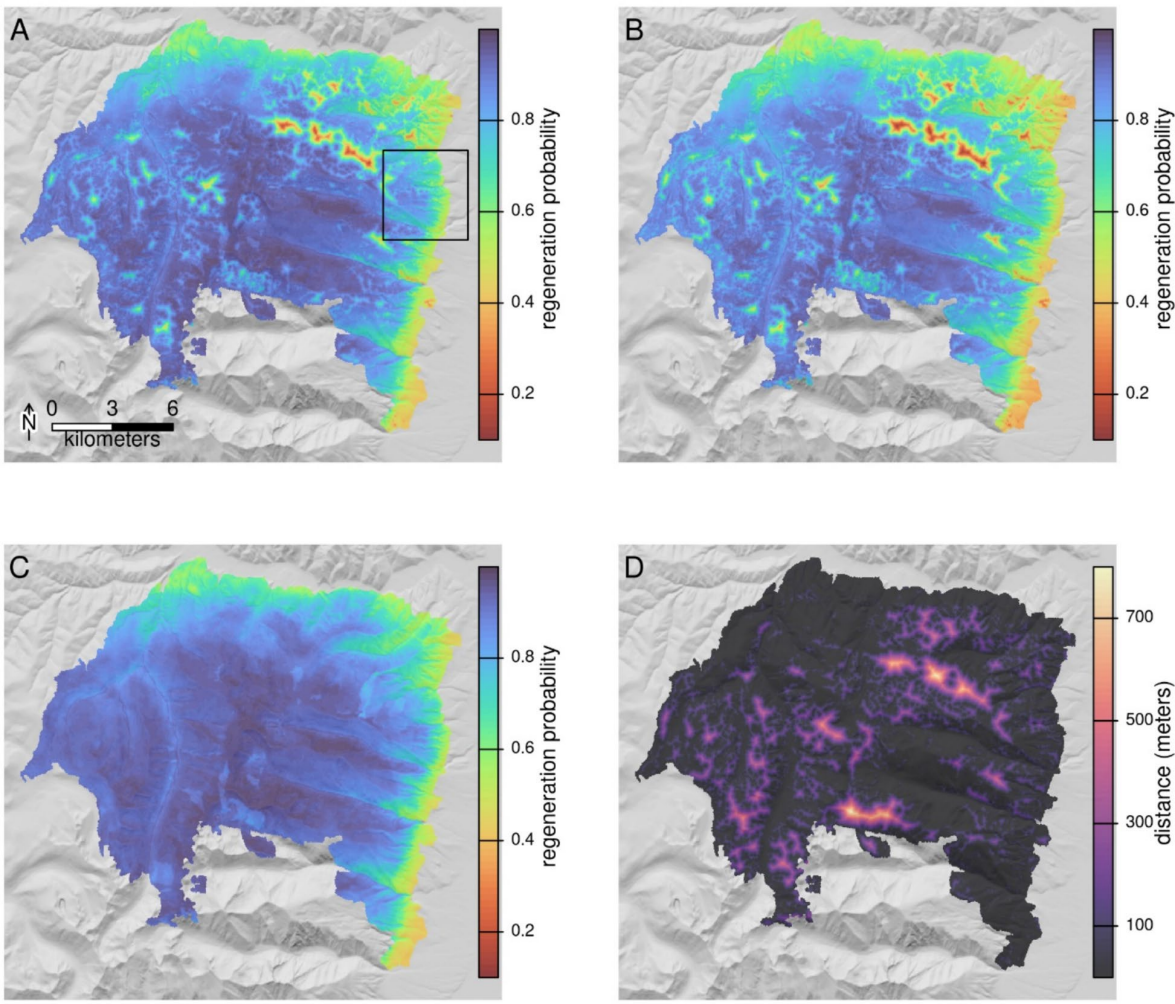
Post-fire regeneration predictions for the 2017 Lolo Peak fire near Florence, Montana. Panel **A** shows the full model prediction, including seed distance effects for the all species model. Panel **B** shows the same model using surface temperature and climatic water deficit projections for 2050. Panel **C** shows the spatial effects of climate in the model, with distance to seed set to a uniform value. Panel **D** shows distance to nearest seed source for context. The black rectangle in panel **A** shows the inset area in Fig. 4

**Fig. 4:**
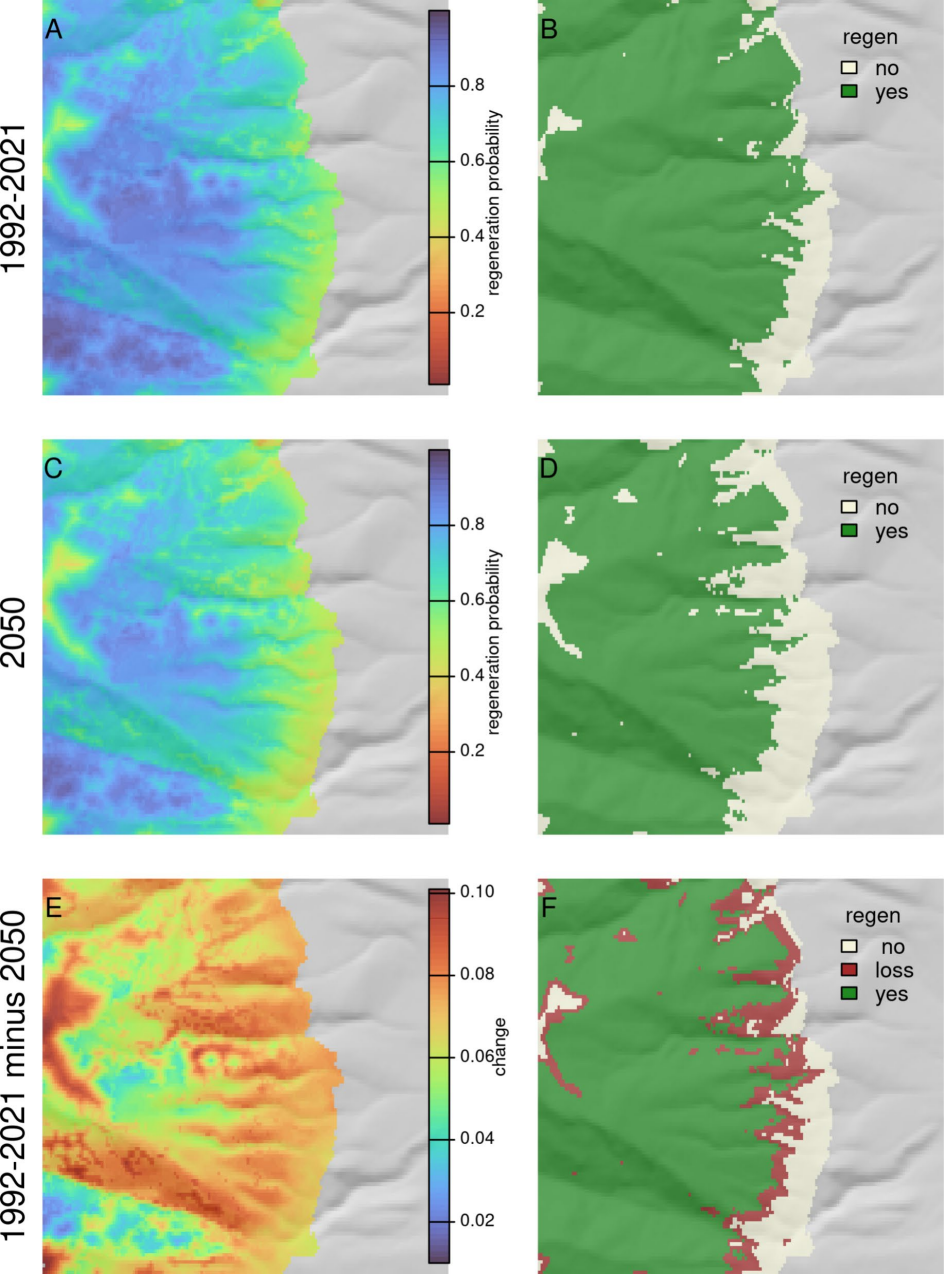
Regeneration probability for a small region of the 2017 Lolo Peak fire near Florence, Montana: **A**&**B**). The top 2 panels show predictions using historical inputs, the bottom panels are for a 2050 average based on 5 GCMs. Panels to the right show regeneration probability classified using 0.6 as a threshold, selected based on maximizing sensitivity and specificity

**Fig. 5:**
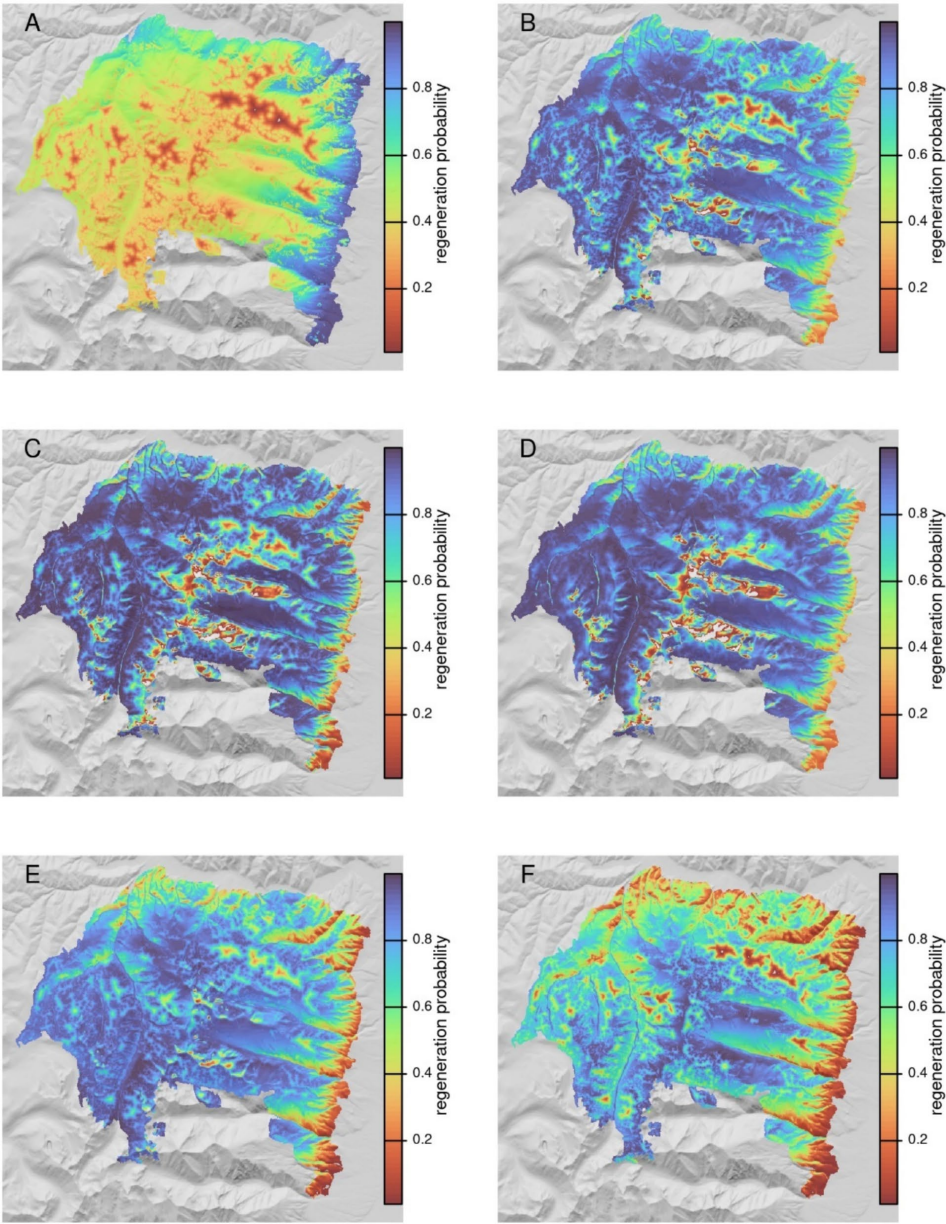
Probability of recruitment model predictions for six conifer species in the 2017 Lolo Peak fire near Florence, Montana: **A** Ponderosa pine; **B** Douglas fir; **C** western larch; **D** lodgepole pine; **E** Engelmann spruce; **F** subalpine fir. The color values for each panel are rescaled using the optimal classification threshold for each species’ model to enable visual comparison

As an example workflow that might be utilized by managers, we show model predictions and Regenmapper outputs for the Lolo Peak fire, which burned roughly 65,000 acres near Florence, Montana in 2017 (Fig. 3). Inset maps for a small area of the fire along the Bitterroot valley are shown in Fig. 4 and spatial predictions for the six individual species’ models are shown in Fig. 5 and Figure S9. For this example, distance to nearest potential live tree was generated using the canopy height layer of Potapov et al. (2021), with 8 m selected as the threshold for meeting seed-producing height, and with both moderate and high-severity classes considered high severity. Patterns of contemporary and future regeneration predictions reveal uphill shifts in area suitable for regeneration over time (Fig. 4). Terrain effects related to aspect and solar insolation are also evident, with future projections illustrating a loss of regeneration potential on some south and west-facing slopes.

**Fig. 6:**
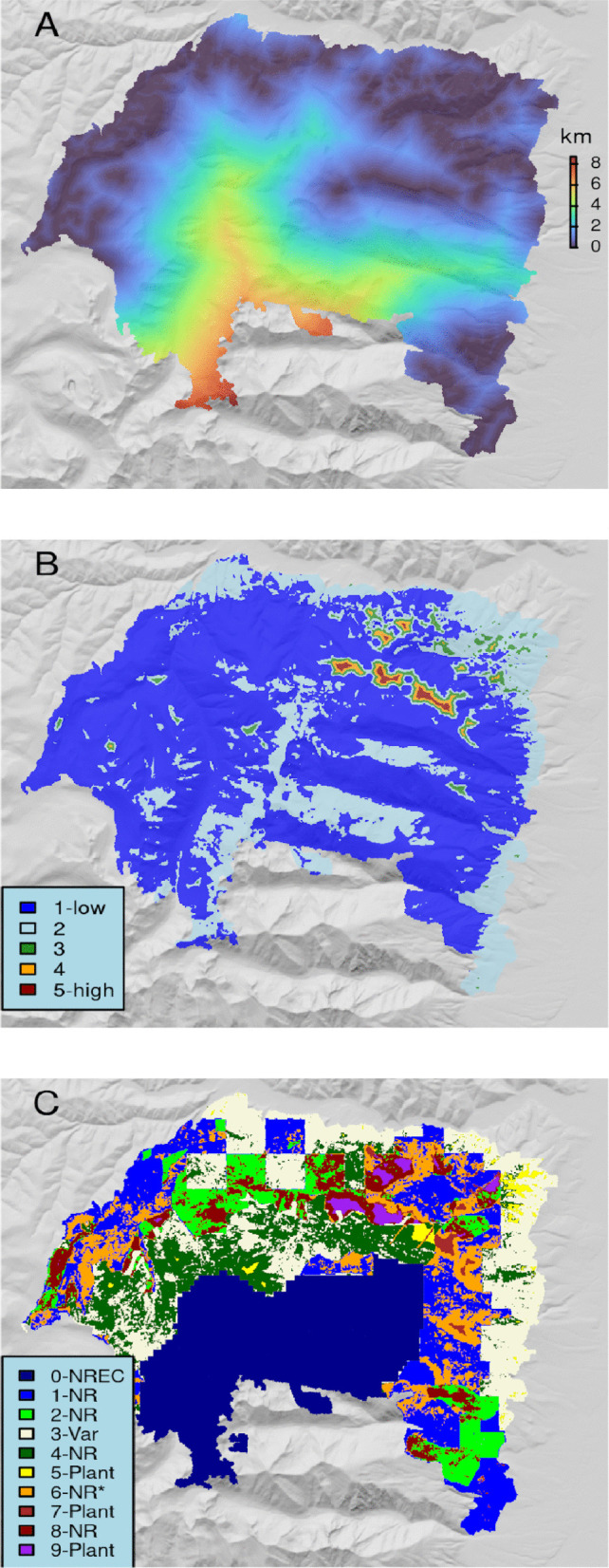
Regenmapper outputs related to prioritization for the 2017 Lolo Peak fire near Florence, Montana: **A** Distance to nearest road in kilometers; **B** 5-class planting prioritization index; **C** USFS administrative prioritization based on the decision matrix in Table 2. Panel **C** shows the final planting diagnosis that includes land management plan considerations. “NREC” means natural recovery in designated wilderness, “NR” means natural regeneration, and “Var” is variable. Classes 8 and 9 are the highest priority classes and occur when existing reforestation needs or incomplete regeneration/final harvests are identified in the USFS FACTS database

Regenmapper outputs designed for prioritization and planning are shown in Fig. 6. Some intuitive patterns are evident in the Planting Prioritization Index (PPI) map: large, high-severity patches with a low probability of natural regeneration that are near roads receive the highest priority (class 5), while areas distant from roads rank low. The administrative prioritization for USFS has broadly similar spatial patterns, but with distinct class boundaries for varying land management designations. The FACTS query for the Lolo Peak fire (accessed Sept. 27th, 2024) returned spatial polygons indicating prior management activities that may trigger reforestation action. These are visible as Class 8 and 9 in Fig. 6.

## Discussion

Regeneration failure following large wildfires is recognized as a threat to sustaining western forests (Coop, et al. 2020). As fire size increases (Iglesias et al. 2022) and short-interval fires increasingly impact western landscapes (Harvey et al. 2023), we expect that more area within wildfire perimeters will be far from surviving trees that can act as seed sources (Buonanduci et al. 2024, 2023). Active reforestation in these areas may be required if recovery and persistence of conifer-dominated forests is the goal. As post-fire tree planting becomes more common, the increased workload limits time for spatial analyses and there is significant potential benefit from a DSS that delivers useful information about post-fire planting conditions. Regenmapper is designed to rapidly deliver actionable information on post-fire planting conditions, providing the best available science in a user-friendly format that can be incorporated in management planning.

The models we developed for predicting post-fire recruitment are consistent with previous studies in showing that distance to and abundance of nearby live trees and climatological mean CWD are strong predictors of regeneration (Fig. 2; Table 2; Stevens-Rumann and Morgan 2019; Stevens-Rumann et al. 2018; Rodman et al. 2020a, b; Davis et al. 2023). We did not include an interaction term between CWD and distance to seed source in our models; however, the difference in dispersal curves between mesic and dry sites (Fig. 2) is noteworthy, as it underscores the fundamental differences in vulnerability between mesic and dry forests, with stronger dispersal constraints in hot, dry areas. Similarly, the interaction between time since fire (how long after a fire sampling occurred) and CWD underscores the potential for divergent responses in dry and mesic areas, with time acting as an exposure term at dry sites and conversely as an agent of opportunity in more mesic sites. Extreme surface temperatures have been linked to seedling mortality in field and laboratory experiments (Kolb and Roberecht 1996; Rank 2022) and previous studies of post-fire conifer regeneration (Davis et al. 2019). In all of our models, PSST was consistently among the strongest predictors of post-fire regeneration (Supp. Tables S3-S9). Together with CWD, this variable provides a useful mechanistic physical template for predicting post-fire recruitment of coniferous tree species. Nevertheless, there are potentially large sources of uncertainty associated with the model inputs, including the threshold selected to define forest presence in the modeled canopy cover data, delayed post-fire mortality of nearby seed sources, and the quality of post-fire burn severity maps (Kolden et al. 2015).

**Table 2:** GLMM output from the combined species model. Covariates were not transformed prior to model fitting, therefore, coefficient estimates are on the scale of each predictor. An asterisk indicates an interaction term between two variables

*Fixed Effects*	*Estimate*	*SE*	*Z-value*	*p*
Intercept	− 0.58	1.21	− 0.48	0.63
Time since fire	0.34	0.04	7.90	0.00
30-year mean annual deficit	0.00	0.00	− 0.83	0.41
Fire severity (RBR)	0.00	0.00	0.39	0.69
Max postfire GS deficit [1st degree]	67.16	26.89	2.50	0.01
Max postfire GS deficit [2nd degree]	− 89.99	25.76	− 3.49	0.00
PSST	− 0.08	0.02	− 3.30	0.00
Distance seed source	− 0.01	0.00	− 13.46	0.00
Clay fraction [1st degree]	− 106.95	23.49	− 4.55	0.00
Clay fraction [2nd degree]	20.08	19.47	1.03	0.30
Surrounding tree cover	0.05	0.00	11.60	0.00
Time since fire*30-year mean annual deficit	0.00	0.00	− 7.37	0.00
Fire severity*Max postfire GS deficit[1st degree]	− 0.06	0.02	− 2.44	0.01
Fire severity*Max postfire GS deficit[2nd degree]	0.01	0.03	0.37	0.71
30-year mean annual deficit* Max postfire GS deficit[1st degree]	− 0.14	0.05	− 2.99	0.00
30-year mean annual deficit* Max postfire GS deficit[2nd degree]	0.11	0.05	2.31	0.02
30-year mean annual deficit* Clay fraction [1st degree]	0.14	0.03	4.28	0.00
30-year mean annual deficit* Clay fraction [2nd degree]	− 0.03	0.03	− 1.06	0.29
*Random effects*				
*σ*^2^	3.29			
*τ*_00 fire_ID_	3.47			
ICC	0.51			
*N*_fire_ID_	322			
Observations	9804			

A number of factors, including seedling production and hiring capacity, could limit the ability to meet large-scale planting objectives (Fargione et al. 2021). Furthermore, new fires each year can add significantly to the land base already slated for planting, making it more difficult to address a backlog of needs in older fires (Dobrowski et al. 2024). These constraints point to a need to prioritize some sites for immediate planting and defer or even abandon others. Regenmapper helps address this need by providing maps of natural regeneration probability and climate suitability for conifer seedlings within specific fire perimeters at a management-relevant spatial resolution. Our prioritization index helps to further refine planting opportunities by integrating these ecological layers with distance to road, a contributor to increased reforestation cost and difficulty (Fargione et al. 2021). There are clear opportunities for maximizing return on investment, for example by avoiding costly plantings in sites that are now too hot or dry for seedlings to survive and instead targeting sites where regeneration without planting is unlikely but climate remains suitable for seedlings. The 5-class prioritization index is intended as a first step toward considering these factors in post-fire planning, but broader-scale analyses aimed at optimizing site selection across fires and regions is a logical next step for improving the DSS.

Planting on land managed by the US Forest Service is often guided by legal and administrative priorities in addition to ecological or optimality constraints. To improve utility and accessibility, we added a decision matrix specific to Forest Service policy considerations and we provide output data in several formats. The decision matrix developed for Forest Service users integrates regeneration probability, previous management information from FACTS, suitable timber layers, where available, and specially designated lands (such as wilderness), for each national forest. The resulting 30-m resolution 10-class priority map assigns the highest priority where reforestation is required by law, but natural regeneration is unlikely, while also highlighting areas like wilderness where management is not allowed. End-users report that raster data and GIS can be barriers for some users, so to maximize utility for a range of user groups, we convert outputs from raster to vector (polygon) format. Users can also upload their own polygon layer (typically representing vegetation units or “stands”) which we attribute with local raster summary values.

### Study limitations

We acknowledge several important limitations and caveats to Regenmapper in its current form that should be considered by users. First, the quality and accuracy of post-fire severity maps used as inputs can have large effects on outputs. Satellite-derived maps of fire severity may only identify the locations of live trees with ca. 80% accuracy (Chapman et al. 2020). In addition, post-fire planting assessments are often made in the months immediately following a fire using whatever cloud-free imagery is available. Consequently, they will not capture effects of delayed mortality, which could lead to optimistic estimates of potential for natural regeneration (Dyer et al. 2025). Users are given the flexibility to define and group different severity classes in maps, but this is nevertheless an issue when assessing severity immediately after a fire. Furthermore, because our “all-species” model includes lodgepole pine (*Pinus contorta*) which is serotinous in some areas, our estimate regeneration in locations that are far from live trees could be optimistic in areas where this species is absent. Conversely, in areas with highly serotinous populations of lodgepole pine, we may underestimate regeneration probability. Nevertheless, a comparison of models with and without lodgepole pine indicated only minor differences in predictions. Currently, Regenmapper provides two maps designed to help prioritize site selection. The first is administrative and specific to US Forest Service users. The second is a simple planting prioritization index (PPI) aimed at identifying severely burned sites that have a low probability of natural regeneration and that are accessible by road. This is a highly simplified index that we consider a starting point for conversations about potential site selection. Spatially explicit optimization methods (e.g., Ager 2024) have been designed for balancing site selection given multiple criteria that could provide more robust estimates of potential planting sites that consider both cost and biophysical constraints.

## Conclusion

Increased investments in reforestation are a unique, once-in-a-generation opportunity to shape the trajectory of forests throughout the western US. Drought, climate change, and wildfires will continue to pose serious challenges and contribute significant uncertainty to how successful we are in regrowing forests over the next decade. Future increases in planting needs will lead to a greater need for landscape prioritization and triage in coming decades and decision support to facilitate these efforts, which can be supported using DSSs such as Regenmapper.

## Supplementary Information

Supplementary Material 1.

## Data Availability

The datasets used in this study are publicly available and described in Table S2. Regenmapper code (regenmapper_v1.2.R) is available through github: https://github.com/topofiredev/regenmapper
