# Supplementary material for: A post-fire reforestation assessment and prioritization tool for the Western United States
Source: Fire Ecol. 2025 Dec 11;21(1):83. doi: 10.1186/s42408-025-00405-z (PMC12698830; doi:10.1186/s42408-025-00405-z)
Supplement: Supplementary file 1 — Supplementary Material 1. [file 42408_2025_405_MOESM1_ESM.pdf]

Supporting Information for:

**A post-fire reforestation assessment and prioritization tool for the Western United States**

[1*]Zachary A. Holden, [2]Ellen Jungck, [1]Kimberley T. Davis, [3]Dyer A. Warren, [3]Alan Swanson, [4]Solomon Dobrowski, [5]Marco Maneta, [6]Kyle C. Rodman, [4]Lewis Faller and [2]Vince Archer

[1*]USDA Forest Service, Rocky Mountain Research Station, Missoula, MT 59808
[2]USDA Forest Service Region 1, Missoula, MT 59808
[3]Department of Community and Public Health, University of Montana, Missoula, MT 59810
[4]Department of Forestry and Conservation, University of Montana, Missoula, MT 59810
[5]Department of Geosciences, University of Montana, Missoula, MT 59810
[6]Ecological Restoration Institute, Northern Arizona University, Flagstaff, AZ 86011, USA

*corresponding author: zachary.holden@usda.gov

This file contains:

Supplementary text S1

Supplementary figures S1-S11 and tables S1-S10

**Text S1. Historical and future hydroclimatic data**

We represent long-term biophysical and climatic conditions at each site using 30-meter resolution 95th percentile annual maximum potential soil surface temperature (PSST) grids (Holden et al. 2024) and a 30-meter climatic water balance deficit (CWD) grid. Links to these datasets are provided in table S2. We developed the CWD layer for the 1992-2021 normal period following methods used to produce PSST grids described by Holden et al. (2024) using time series of daily weather data (temperature, relative humidity, solar radiation, wind speed, precipitation) generated at a network of ~61,000 randomly selected points. These datasets were previously used to map PSST and additional details on the point sampling distribution and mapping methods can be found in Holden et al. (2024). This point-based approach avoids the massive computation required to simulate changes across the relatively dense 8 arc-second (~250 meter) resolution grids, while retaining terrain-resolving features of the gridded datasets. At each of 61,000 sample points, we simulated the daily water balance from 1992-2021 with a snow and soil moisture model, using weather inputs (temperature, humidity, radiation, wind speed) extracted from 250-meter resolution daily grids (Holden et al. 2018). We then use data from the 6th coupled model intercomparison project (CMIP6; Eyring et al. 2016) and a stochastic weather simulator (Steinscheider and Brown 2013) to generate daily weather inputs for the 2050 mean period (2035-2065) at each sample point, using monthly mean deviations in temperature and precipitation based on five GCM's (Table S1). For our target variable (climate water deficit--potential minus actual evapotranspiration), we calculated the climatological average value for the historical (1992-2021) and future (2050) mean at each point and then interpolated those values using geographically weighted regression. This point-based modeling approach allows for gridding the water balance outputs at a relatively fine 30-meter spatial resolution, which would be computationally intractable when applying the same algorithms to high resolution grids.

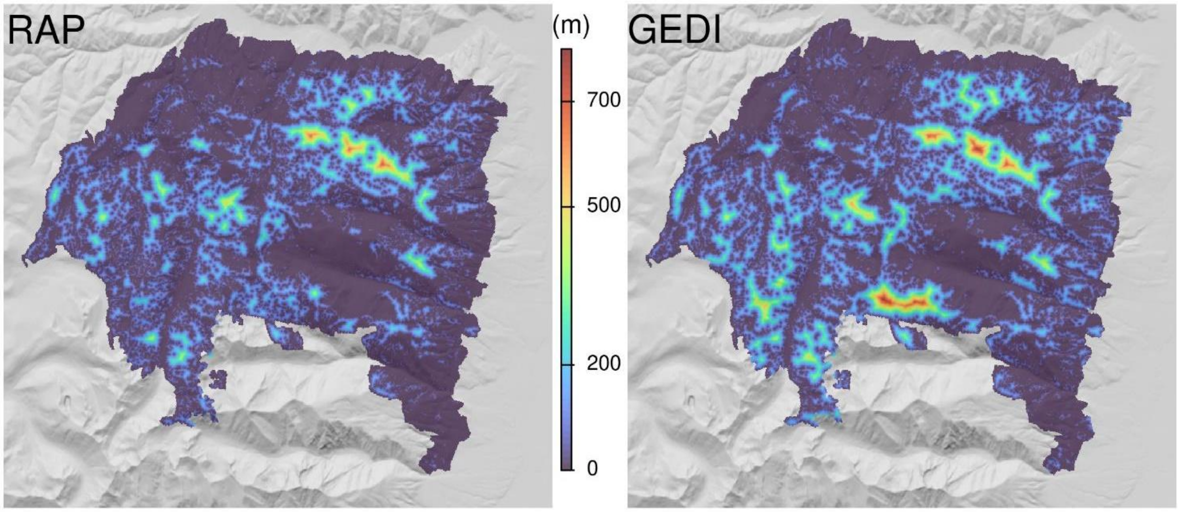


Figure S1. Distance to seed source estimated for the Lolo Peak fire using pre-fire (2016) RAP forest canopy cover (left) and GEDI canopy height (right).

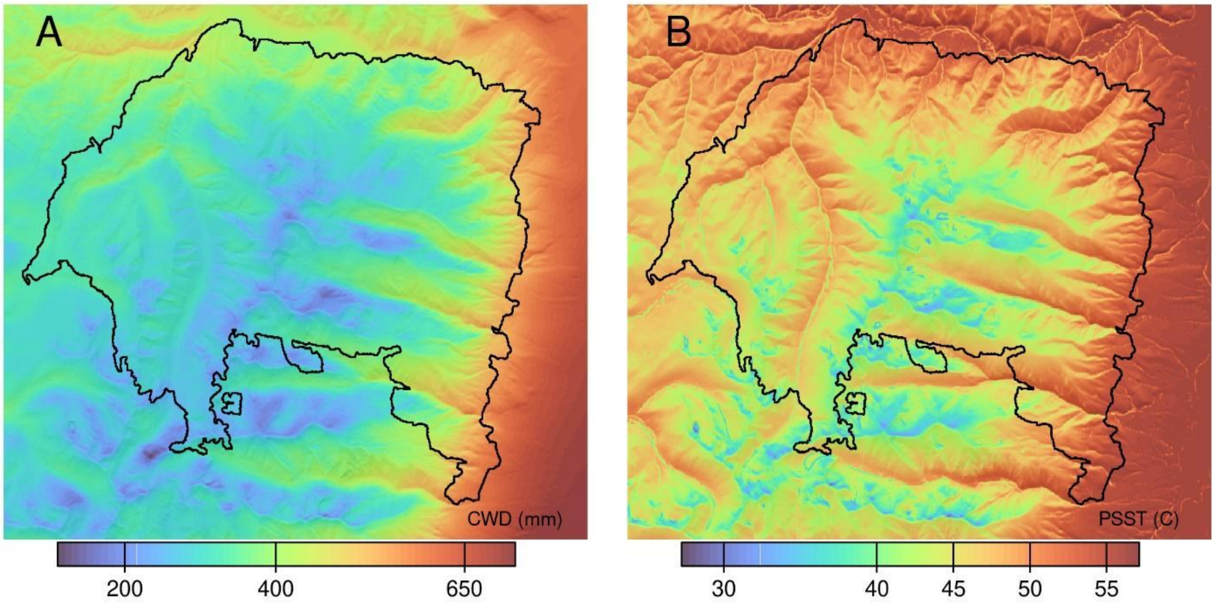


Figure S2. Climatic predictors used in regression models predicting post-fire regeneration. A) 1992-2021 climatic water balance deficit and B) potential soil surface temperature.

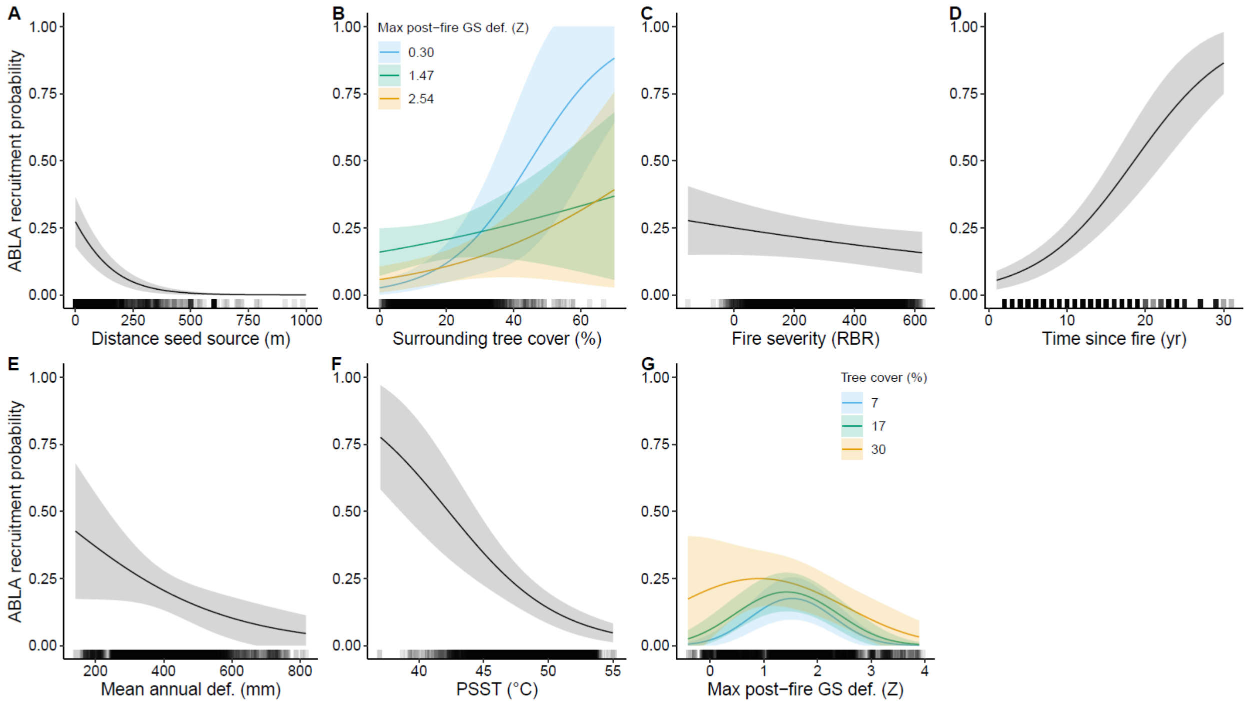


Figure S3. Partial dependence plots for the *Abies lasiocarpa* model showing relationship between model predictors and post-fire recruitment while holding other variables constant at their medians. The interaction is shown between surrounding tree cover and maximum post-fire growing season water deficit ("def."; max within the first five post-fire years) by plotting lines for the $10^{th}$, $50^{th}$, and $90^{th}$ percentile values of the interacting variable from the dataset used to create the model. Rug plot on the x-axes show the distribution of data.

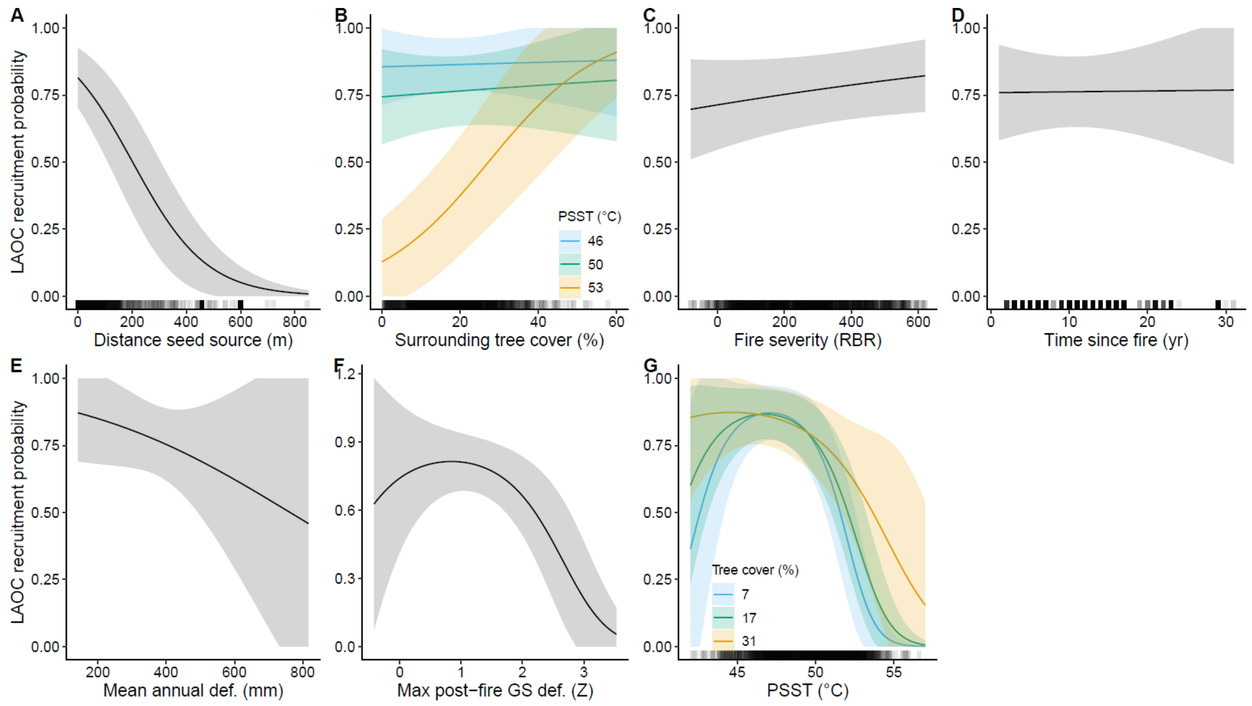


Figure S4. Partial dependence plots for the *Larix occidentalis* model showing relationship between model predictors and post-fire recruitment while holding other variables constant at their medians. The interaction is shown between surrounding tree cover and potential surface skin temperature ("PSST") by plotting lines for the 10$^{th}$, 50$^{th}$, and 90$^{th}$ percentile values of the interacting variable from the dataset used to create the model. Rug plot on the x-axes show the distribution of data.

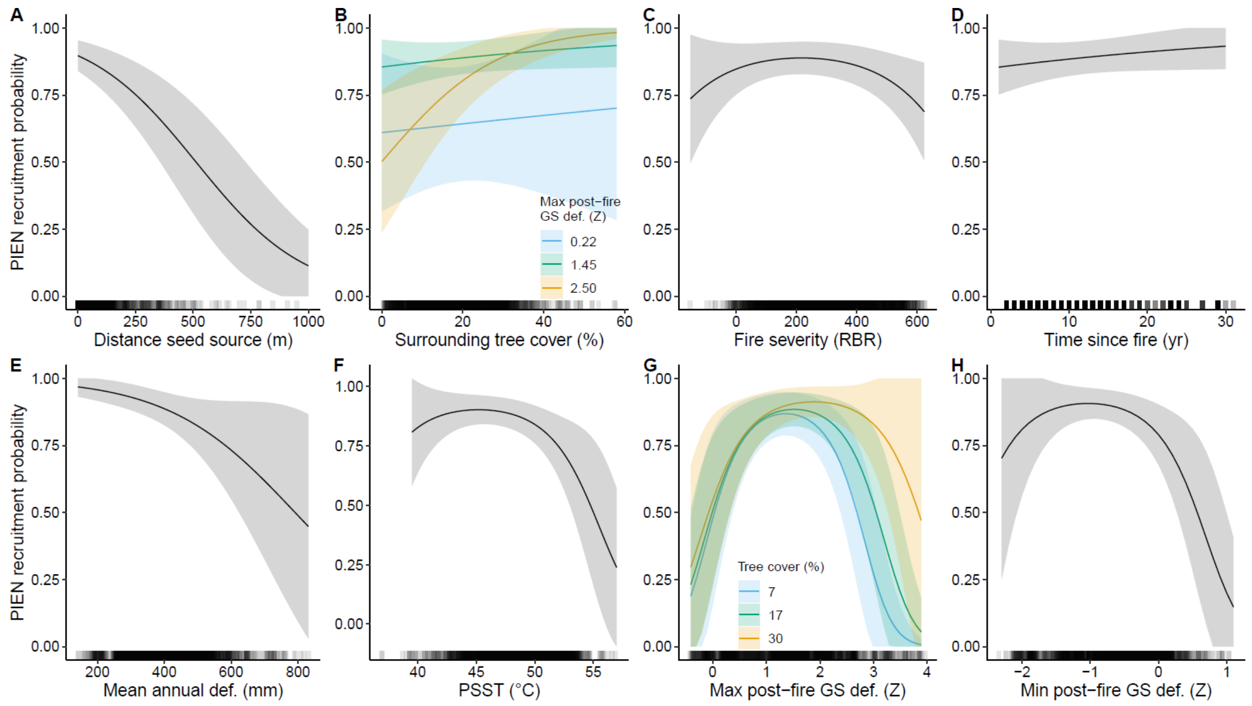


Figure S5. Partial dependence plots for the *Picea engelmannii* model showing relationship between model predictors and post-fire recruitment while holding other variables constant at their medians. The interaction is shown between surrounding tree cover and maximum post-fire growing season water deficit ("def."; max within the first five post-fire years) by plotting lines for the 10$^{th}$, 50$^{th}$, and 90$^{th}$ percentile values of the interacting variable from the dataset used to create the model. Rug plot on the x-axes show the distribution of data.

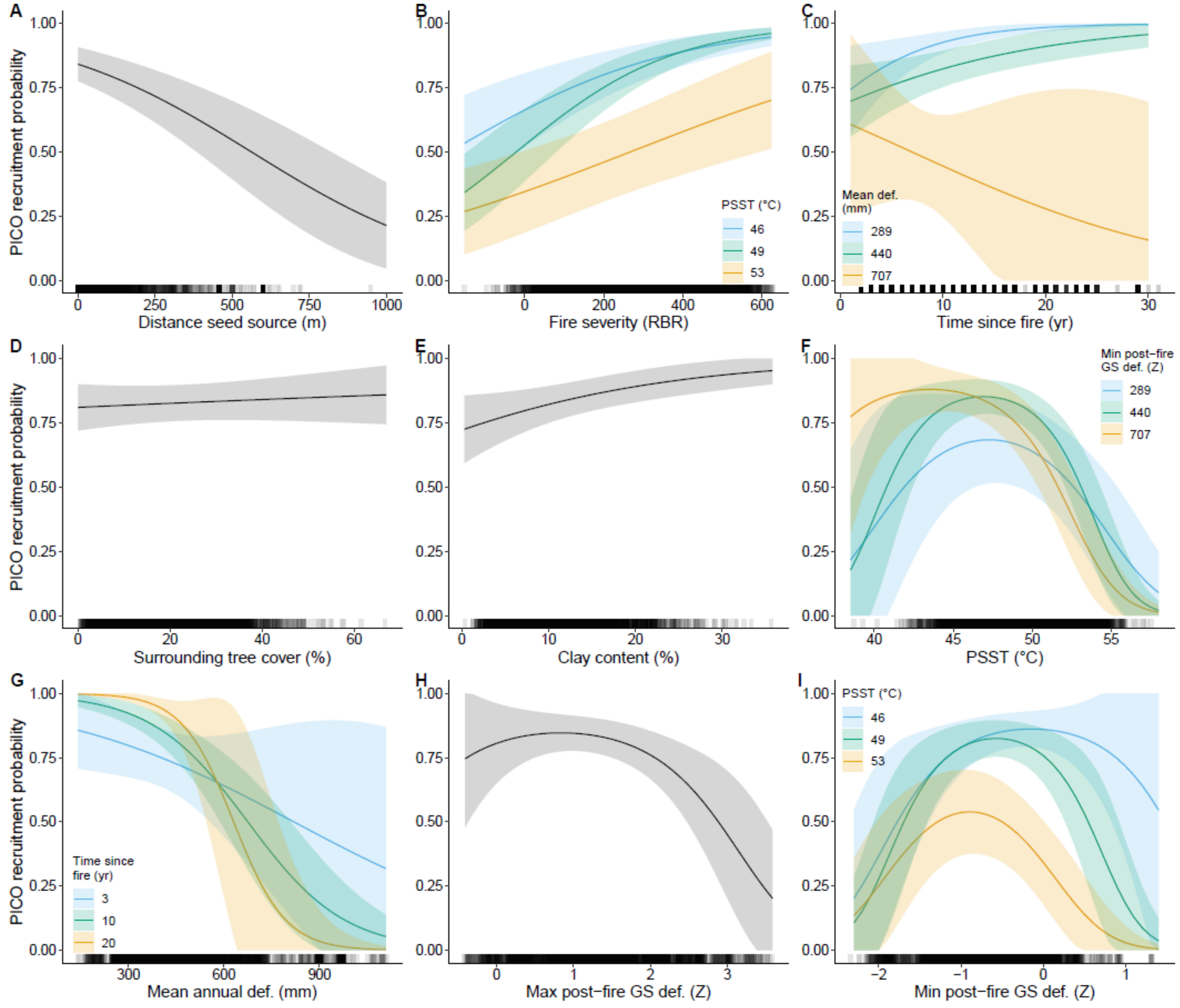


Figure S6. Partial dependence plots for the *Pinus contorta* model showing relationship between model predictors and post-fire regeneration while holding other variables constant at their medians. Interactions between variables are shown by plotting lines for the 10$^{th}$, 50$^{th}$, and 90$^{th}$ percentile values of the interacting variable from the dataset used to create the model. Rug plots on the x-axes show the distribution of data.

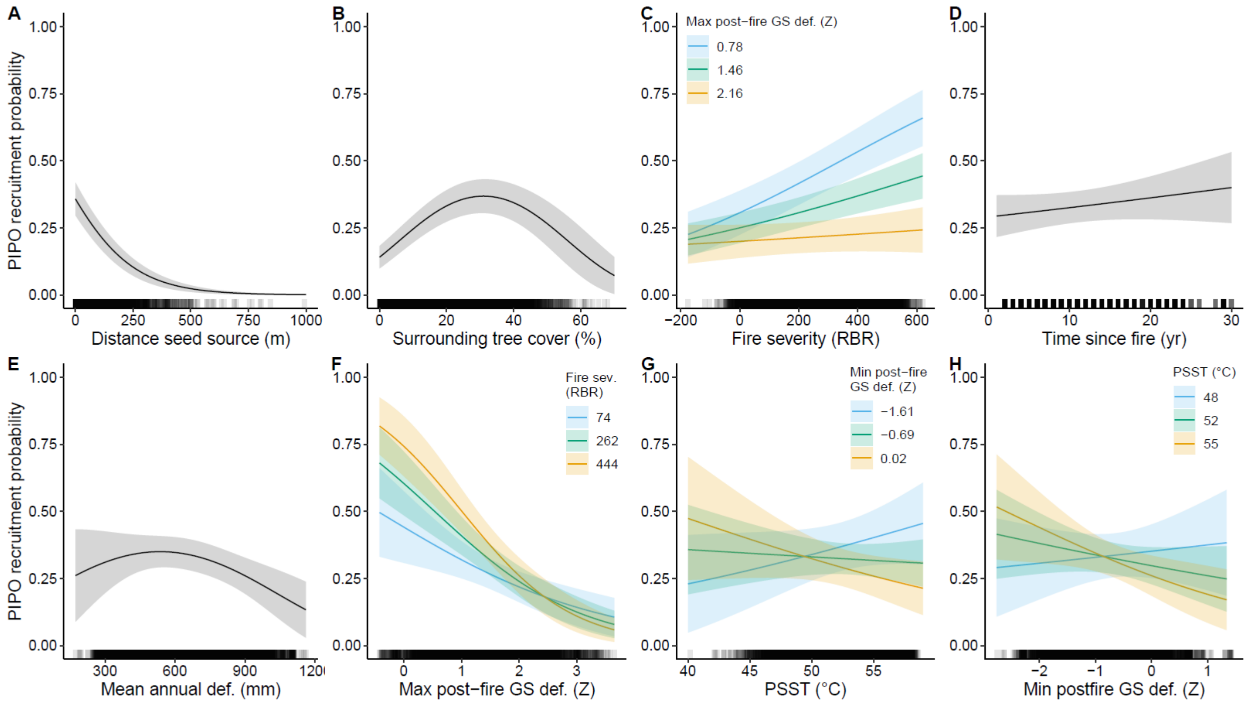


Figure S7. Partial dependence plots for the *Pinus ponderosa/P. jeffreyi* model showing relationship between model predictors and post-fire regeneration while holding other variables constant at their medians. The interactions between RBR and maximum post-fire growing season water deficit ("def."; max within the first 5 post-fire years) and potential surface skin temperature ("PSST") and minimum post-fire growing season water deficit (min within the first 5 post-fire years) are shown by plotting lines for the 10$^{th}$, 50$^{th}$, and 90$^{th}$ percentile values of the interacting variable from the dataset used to create the model. Rug plots on the x-axes show the distribution of data.

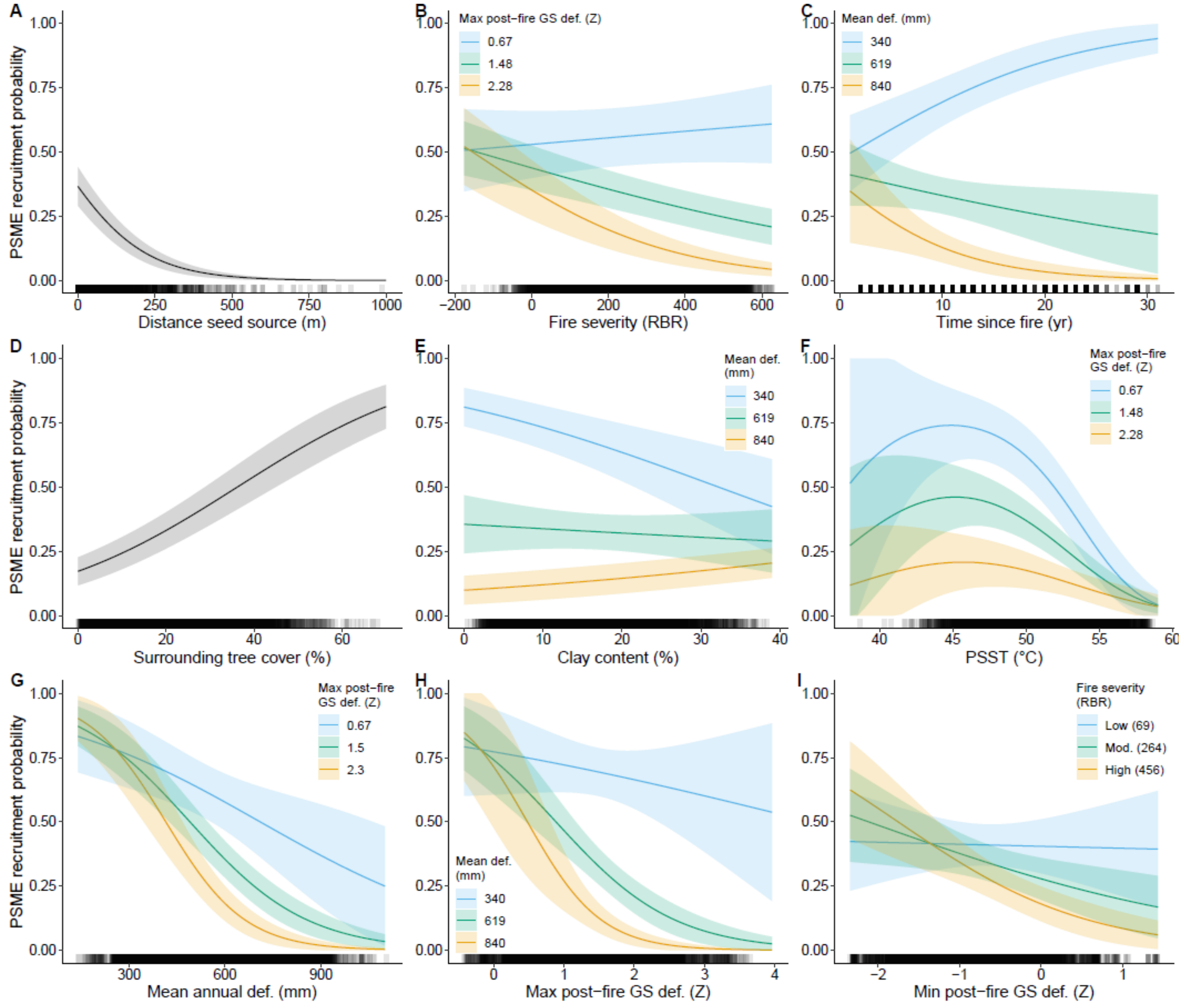


Figure S8. Partial dependence plots for the *Pseudotsuga menziesii* model showing relationship between model predictors and post-fire regeneration while holding other variables constant at their medians. The interactions between fire severity (RBR) and post-fire maximum growing season water deficit ("def"; max/min within the first five post-fire years), potential surface skin temperature ("PSST") and 30-year mean annual water deficit, and 30-year mean annual water deficit and post-fire maximum growing season water deficit are shown by plotting lines for the 10$^{th}$, 50$^{th}$, and 90$^{th}$ percentile values of the interacting variable from the dataset used to create the model. Rug plots on the x-axes show the distribution of data.

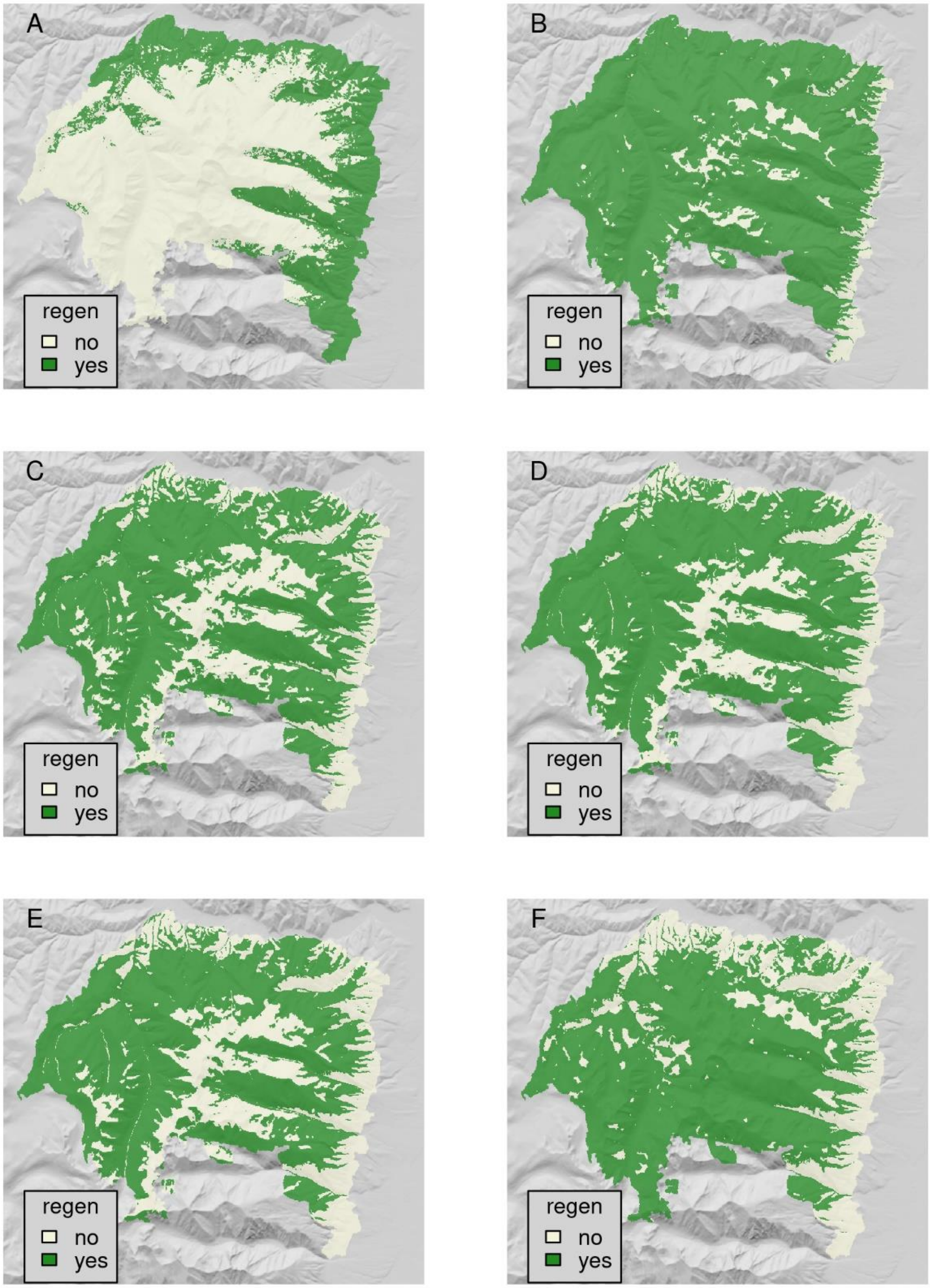


Figure S9. A companion to Figure 5 showing recruitment probability for six conifers species, classified into a binary outcome: A) Ponderosa pine; B) Douglas fir; C) western larch; D) lodgepole pine; E) Engelmann spruce; F) subalpine fir. The threshold values for each panel was determined by optimizing sensitivity plus specificity and are shown in Table S8.

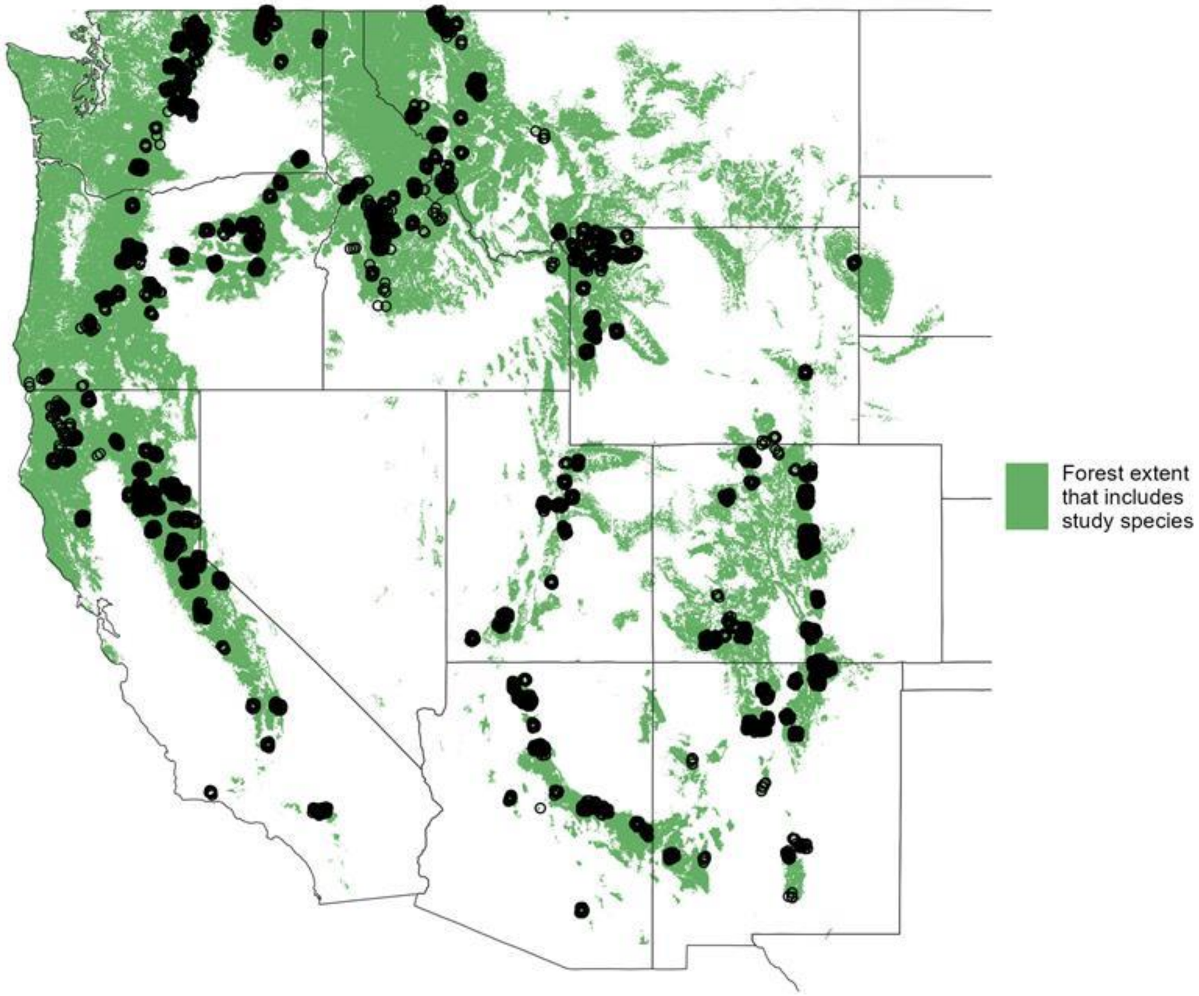


Figure S10. Map showing the locations of field plots used in this study.

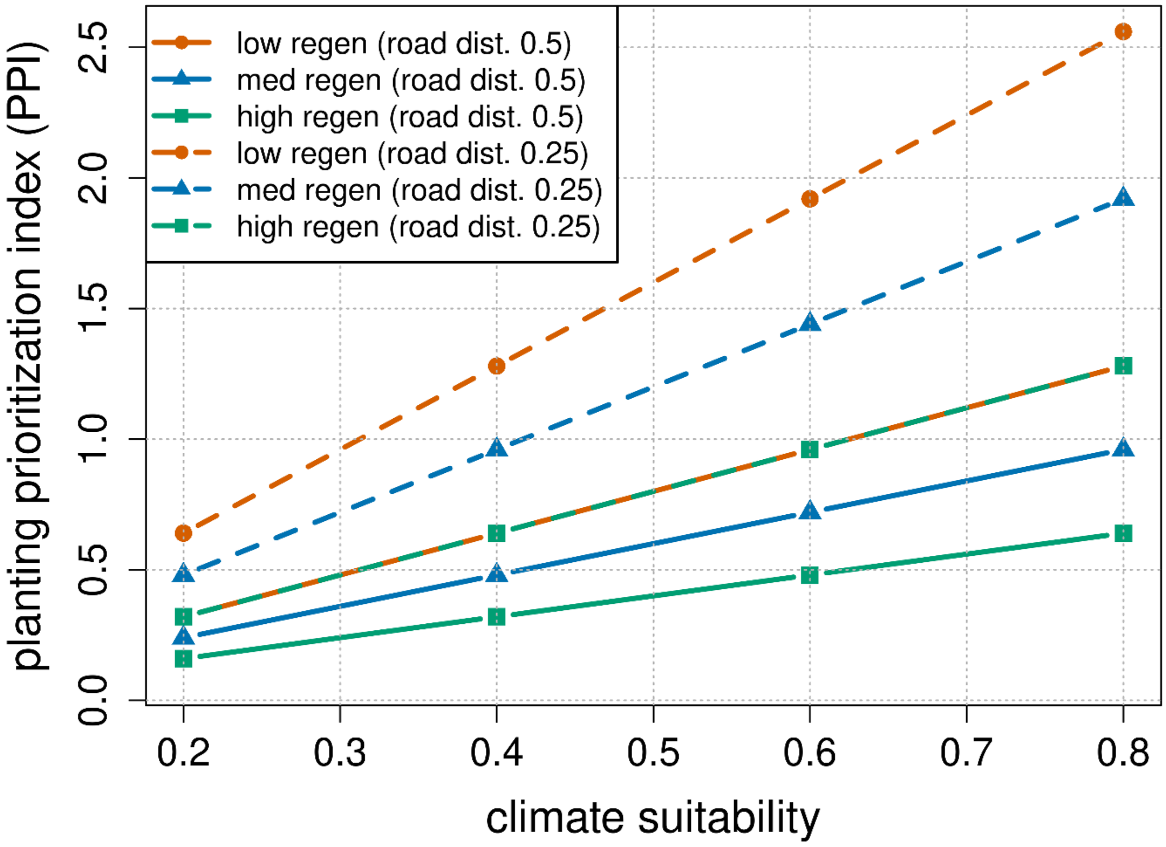


Figure S11. Planting prioritization index values (PPI) plotted combinations of regeneration probability, climatic suitability and road distance values. Regeneration probability values are held at 0.2 (low), 0.4 (medium) and 0.6 (high) values and Road distances are 0.25 and 0.5 kilometers. Higher values (higher priority) occur where natural regeneration probability is low but climatic suitability is high, indicating sites with dispersal constraints where planted trees are likely to survive.

Table S1. Five GCMs used to represent future climate conditions for the US.

| Climate model | institution |
|---|---|
| MIROC6 | JAMSTEC (Japan Agency for Marine-Earth Science and Technology), AORI (Atmosphere and Ocean Research Institute), NIES (National Institute for Environmental Studies), and R-CCS (RIKEN Center for Computational Science) (Japan) |
| MPI-ESM1-2-LR | Max Planck Institute for Meteorology (Germany) |
| GISS-E2-1-G | Goddard Institute for Space Studies (USA) |
| CNRM-ESM2 | National Centre of Meteorological Research, France |
| EC-Earth3 | EC-Earth Consortium (European Community) |

Table S2. Locations for downloading datasets used in this study.

| Dataset | Description | Source |
|---|---|---|
| Plot data | Post-fire conifer presence/absence data | https://doi.org/10.5061/dryad.0rxwdbs47 |
| PSST | 30-meter resolution Potential soil surface temperature grids | https://topofire.dbs.umt.edu/public_data/PSST/ |
| CWD | 30-meter resolution climatic water deficit raster grid | https://topofire.dbs.umt.edu/public_data/topofire_weather/normals |
| Topofire daily weather | Daily 8 arc sec weather and soil water balance grids stored as monthly ncdf files | https://topofire.dbs.umt.edu/public_data/topofire_weather/daily_grids/ |

Table S3. Model output from the *Abies lasiocarpa* model.

| | ABLA Model | | | |
|---|---|---|---|---|
| *Predictors* | *Log-Odds* | *std. Error* | *Statistic* | *p* |
| Intercept | 4.952 | 1.957 | 2.530 | **0.011** |
| Time since fire | 0.162 | 0.024 | 6.774 | **<0.001** |
| 30-yr mean annual deficit | -0.004 | 0.002 | -2.225 | **0.026** |
| Surrounding tree cover | 0.038 | 0.010 | 3.608 | **<0.001** |
| Distance seed source | -0.008 | 0.001 | -8.303 | **<0.001** |
| Fire severity (RBR) | -0.001 | 0.001 | -1.701 | 0.089 |
| Max postfire GS deficit [1st degree] | -2.754 | 12.122 | -0.227 | 0.820 |
| Max postfire GS deficit [2nd degree] | -55.558 | 12.608 | -4.407 | **<0.001** |
| PSST | -0.236 | 0.047 | -5.041 | **<0.001** |
| Surrounding tree cover*Max postfire GS deficit [1st degree] | -0.367 | 0.443 | -0.828 | 0.407 |
| Surrounding tree cover*Max postfire GS deficit [2nd degree] | 1.455 | 0.457 | 3.181 | **0.001** |
| **Random Effects** | | | | |
| $\sigma^2$ | 3.29 | | | |
| $\tau_{00\ \mathrm{fire\_name}}$ | 3.12 | | | |
| ICC | 0.49 | | | |
| $N_{\mathrm{fire\_name}}$ | 139 | | | |
| Observations | 2178 | | | |
| Marginal $R^2$ / Conditional $R^2$ | 0.456 / 0.720 | | | |

Table S4. Model output from the *Larix occidentalis* model.

| | LAOC Model | | | |
|---|---|---|---|---|
| *Predictors* | *Log-Odds* | *std. Error* | *Statistic* | *p* |
| Intercept | -3.129 | 1.324 | -2.363 | **0.018** |
| Time since fire | 0.002 | 0.035 | 0.049 | 0.961 |
| Fire severity (RBR) | 0.001 | 0.001 | 1.276 | 0.202 |
| 30-yr mean annual deficit | -0.003 | 0.003 | -1.041 | 0.298 |
| Max postfire GS deficit [1st degree] | -18.404 | 11.116 | -1.656 | 0.098 |
| Max postfire GS deficit [2nd degree] | -19.121 | 7.492 | -2.552 | **0.011** |
| PSST [1st degree] | -42.133 | 10.861 | -3.879 | **<0.001** |
| PSST [2nd degree] | -33.068 | 10.023 | -3.299 | **0.001** |
| Surrounding tree cover | 0.021 | 0.013 | 1.601 | 0.109 |
| Distance seed source | -0.007 | 0.001 | -7.576 | **<0.001** |
| Surrounding tree cover*PSST [1st degree] | 0.726 | 0.409 | 1.773 | 0.076 |
| Surrounding tree cover*PSST [2nd degree] | 0.849 | 0.445 | 1.906 | 0.057 |
| **Random Effects** | | | | |
| $\sigma^2$ | 3.29 | | | |
| $\tau_{00\ fire\_name}$ | 6.65 | | | |
| ICC | 0.67 | | | |
| $N_{fire\_name}$ | 84 | | | |
| Observations | 1157 | | | |
| Marginal $R^2$ / Conditional $R^2$ | 0.316 / 0.774 | | | |

Table S5. Model output from *Picea engelmannii* model.

| | PIEN Model | | | |
|---|---|---|---|---|
| *Predictors* | *Log-Odds* | *std. Error* | *Statistic* | *p* |
| Intercept | -2.430 | 0.923 | -2.634 | **0.008** |
| Time since fire | 0.029 | 0.031 | 0.957 | 0.338 |
| 30-yr mean annual deficit | -0.005 | 0.002 | -2.730 | **0.006** |
| Distance seed source | -0.004 | 0.001 | -6.406 | **<0.001** |
| Fire severity (RBR) [1st degree] | -7.024 | 3.755 | -1.871 | 0.061 |
| Fire severity (RBR) [2nd degree] | -8.021 | 3.124 | -2.567 | **0.010** |
| PSST [1st degree] | -14.560 | 6.297 | -2.312 | **0.021** |
| PSST [2nd degree] | -11.706 | 4.552 | -2.572 | **0.010** |
| Surrounding tree cover | 0.030 | 0.012 | 2.466 | **0.014** |
| Max postfire GS deficit [1st degree] | -21.536 | 11.917 | -1.807 | 0.071 |
| Max postfire GS deficit [2nd degree] | -48.103 | 12.851 | -3.743 | **<0.001** |
| Min postfire GS deficit [1st degree] | -14.421 | 8.492 | -1.698 | 0.089 |
| Min postfire GS deficit [2nd degree] | -15.556 | 5.747 | -2.707 | **0.007** |
| Surrounding tree cover*Max postfire GS deficit [1st degree] | 1.155 | 0.425 | 2.718 | **0.007** |
| Surrounding tree cover*Max postfire GS deficit [2nd degree] | 0.792 | 0.479 | 1.655 | 0.098 |
| **Random Effects** | | | | |
| $\sigma^2$ | 3.29 | | | |
| $\tau_{00\ fire\_name}$ | 4.12 | | | |
| ICC | 0.56 | | | |
| $N_{fire\_name}$ | 138 | | | |
| Observations | 1514 | | | |
| Marginal $R^2$ / Conditional $R^2$ | 0.301 / 0.689 | | | |

Table S6. Model output from the *Pinus contorta* model.

| | PICO Model | | | |
|---|---|---|---|---|
| *Predictors* | *Log-Odds* | *std. Error* | *Statistic* | *p* |
| Intercept | -6.0044 | 1.1791 | -5.0922 | **<0.001** |
| Time since fire | 0.3277 | 0.1170 | 2.8015 | **0.005** |
| 30-yr mean annual deficit | -0.0009 | 0.0026 | -0.3589 | 0.720 |
| Surrounding tree cover | 0.0053 | 0.0086 | 0.6154 | 0.538 |
| Distance seed source | -0.0030 | 0.0005 | -5.9616 | **<0.001** |
| Fire severity (RBR) | 0.0037 | 0.0005 | 7.9307 | **<0.001** |
| PSST [1st degree] | -31.8384 | 8.7145 | -3.6535 | **<0.001** |
| PSST [2nd degree] | 7.2747 | 7.3576 | 0.9887 | 0.323 |
| Soil clay content (%) | 0.0573 | 0.0217 | 2.6429 | **0.008** |
| Min post-fire GS deficit [1st degree] | 9.5945 | 9.6683 | 0.9924 | 0.321 |
| Min post-fire GS deficit [2nd degree] | -26.0409 | 6.6599 | -3.9101 | **<0.001** |
| Max post-fire GS deficit [1st degree] | -19.1755 | 9.2589 | -2.0710 | **0.038** |
| Max post-fire GS deficit [2nd degree] | -18.3139 | 7.4727 | -2.4508 | **0.014** |
| 30-yr mean annual deficit*Time since fire | -0.0006 | 0.0003 | -1.9684 | **0.049** |
| Fire severity (RBR)*PSST [1st degree] | -0.0177 | 0.0238 | -0.7429 | 0.458 |
| Fire severity (RBR)*PSST [2nd degree] | -0.0951 | 0.0218 | -4.3626 | **<0.001** |
| Min post-fire GS deficit [1st degree]*PSST [1st degree] | -892.1663 | 302.2709 | -2.9515 | **0.003** |
| Min post-fire GS deficit [1st degree]*PSST [2nd degree] | -90.6521 | 224.8927 | -0.4031 | 0.687 |
| Min post-fire GS deficit [2nd degree]*PSST [1st degree] | -253.3524 | 271.3187 | -0.9338 | 0.350 |
| Min post-fire GS deficit [2nd degree]*PSST [2nd degree] | 528.5719 | 233.3446 | 2.2652 | **0.024** |
| **Random Effects** | | | | |
| $\sigma^2$ | 3.29 | | | |
| $\tau_{00\ fire\_name}$ | 4.97 | | | |
| ICC | 0.60 | | | |
| $N_{fire\_name}$ | 181 | | | |
| Observations | 3252 | | | |

| | |
|---|---|
| Marginal $R^2$ / Conditional $R^2$ | 0.330 / 0.733 |

Table S7. Model output from the *Pinus ponderosa/P. jeffreyi* model.

| | **PIPO Model** | | | |
|---|---|---|---|---|
| *Predictors* | *Log-Odds* | *std. Error* | *Statistic* | *p* |
| Intercept | -2.4120 | 1.9522 | -1.2356 | 0.217 |
| Time since fire | 0.0162 | 0.0132 | 1.2278 | 0.220 |
| 30-yr mean annual deficit [1st degree] | -11.5804 | 7.8667 | -1.4721 | 0.141 |
| 30-yr mean annual deficit [2nd degree] | -10.9197 | 5.9673 | -1.8299 | 0.067 |
| Surrounding tree cover [1st degree] | 22.0147 | 4.1017 | 5.3673 | **<0.001** |
| Surrounding tree cover [2nd degree] | -19.0400 | 3.2252 | -5.9035 | **<0.001** |
| Distance seed source | -0.0063 | 0.0005 | -12.1275 | **<0.001** |
| Fire severity (RBR) | 0.0041 | 0.0008 | 4.8484 | **<0.001** |
| Max postfire GS deficit | -0.4135 | 0.1773 | -2.3322 | **0.020** |
| Min postfire GS deficit | 3.3272 | 1.6638 | 1.9998 | **0.046** |
| PSST | -0.0616 | 0.0358 | -1.7227 | 0.085 |
| Fire severity*Max postfire GS deficit | -0.0014 | 0.0004 | -3.2834 | **0.001** |
| Fire severity*Min postfire GS deficit | 0.0009 | 0.0004 | 2.0706 | **0.038** |
| PSST*Min postfire GS deficit | -0.0719 | 0.0318 | -2.2631 | **0.024** |
| **Random Effects** | | | | |
| $\sigma^2$ | 3.29 | | | |
| $\tau_{00}$ fire_name | 1.86 | | | |
| ICC | 0.36 | | | |
| N fire_name | 264 | | | |
| Observations | 7342 | | | |
| Marginal $R^2$ / Conditional $R^2$ | 0.138 / 0.450 | | | |

Table S8. Model output from *Pseudotsuga menziesii* model.

| | **PSME Model** | | | |
|---|---|---|---|---|
| *Predictors* | *Log-Odds* | *std. Error* | *Statistic* | *p* |
| Intercept | -5.9872 | 1.3577 | -4.4099 | **<0.001** |
| Time since fire | 0.2541 | 0.0482 | 5.2766 | **<0.001** |
| 30-yr mean annual deficit | 0.0022 | 0.0023 | 0.9685 | 0.333 |
| Surrounding tree cover | 0.0434 | 0.0049 | 8.8241 | **<0.001** |
| Distance seed source | -0.0072 | 0.0007 | -10.9017 | **<0.001** |
| Fire severity | 0.0012 | 0.0010 | 1.2143 | 0.225 |
| Max postfire GS deficit | 1.6466 | 0.6146 | 2.6793 | **0.007** |
| Min postfire GS deficit | 0.1156 | 0.2316 | 0.4990 | 0.618 |
| Clay | -0.0910 | 0.0398 | -2.2850 | **0.022** |
| PSST [1st degree] | -73.4875 | 17.8468 | -4.1177 | **<0.001** |
| PSST [2nd degree] | -22.7615 | 10.9509 | -2.0785 | **0.038** |
| Time since fire*30-yr mean annual deficit | -0.0005 | 0.0001 | -5.6805 | **<0.001** |
| Fire severity*Max postfire GS deficit | -0.0028 | 0.0005 | -5.4633 | **<0.001** |
| Fire severity*Min postfire GS deficit | -0.0021 | 0.0005 | -4.3976 | **<0.001** |
| 30-yr mean annual deficit*Max postfire GS deficit | -0.0033 | 0.0011 | -3.0565 | **0.002** |
| 30-yr mean annual deficit*Clay | 0.0001 | 0.0001 | 2.1800 | **0.029** |
| 30-yr mean annual deficit*PSST [1st degree] | 20.1167 | 10.9371 | 1.8393 | 0.066 |
| 30-yr mean annual deficit*PSST [2nd degree] | 5.4957 | 6.5936 | 0.8335 | 0.405 |
| **Random Effects** | | | | |
| $\sigma^2$ | 3.29 | | | |
| $\tau_{00\ fire\_name}$ | 4.02 | | | |
| ICC | 0.55 | | | |
| $N_{fire\_name}$ | 273 | | | |
| Observations | 5964 | | | |
| Marginal $R^2$ / Conditional $R^2$ | 0.320 / 0.694 | | | |

Table S9. Model performance metrics for each species and the all-species models. "AUC" refers to the area under the receiver operating characteristic curve. "CV AUC" is the mean AUC from 10-fold cross validation (predictions made to data not used to build the model, all plots from a single fire are in the same fold). "Threshold$^{ss}$" refers to the probability threshold used to categorize regeneration as likely or unlikely which maximizes the sum of specificity and sensitivity (26). "Threshold$^{k}$" refers to the probability threshold used to categorize regeneration as likely or unlikely which maximizes Cohen's kappa (27).

| Species | Plots | Fires | AUC | CV AUC | Threshold$^{ss}$ | Threshold$^{k}$ |
|---|---|---|---|---|---|---|
| *Pinus ponderosa/P. jeffreyi* | 7342 | 264 | 0.70 | 0.69 | 0.29 | 0.32 |
| *Pseudotsuga menziesii* | 5964 | 273 | 0.73 | 0.73 | 0.34 | 0.34 |
| *Larix occidentalis* | 1157 | 84 | 0.74 | 0.67 | 0.37 | 0.36 |
| *Pinus contorta* | 3252 | 181 | 0.78 | 0.76 | 0.56 | 0.45 |
| *Picea engelmannii* | 1514 | 138 | 0.74 | 0.67 | 0.50 | 0.50 |
| *Abies lasiocarpa* | 2178 | 139 | 0.79 | 0.77 | 0.16 | 0.33 |
| *Combined species* | 9804 | 322 | 0.75 | 0.74 | 0.60 | 0.59 |

Table S10. Activity codes from the U.S. Forest Service activity database (FACTS) that indicate a previously existing reforestation need. These codes receive higher priorityin the administrative prioritization of planting sites shown in figure 6C.

| FACTS Activity Code | Description |
|---|---|
| 4101 | Coppice cut |
| 4102 | Coppice Cut (w/leave trees) |
| 4111 | Patch clearcutting |
| 4113 | Stand Clearcut |
| 4115 | Patch clearcutting (w/ leave trees) |
| 4117 | Stand clearcutting (w/ leave trees) |
| 4121 | Shelterwood preparation cut |
| 4122 | Seed-tree Preparatory Cut |
| 4131 | Shelterwood Establishment Cut (with or without leave trees) |
| 4132 | Seed-tree Seed Cut (with and without leave trees) |
| 4141 | Shelterwood Removal Cut |
| 4142 | Seed-tree Final Cut |
| 4143 | Overstory Removal Cut (from advanced regeneration) |
| 4145 | Shelterwood Removal Cut (w/ leave trees) |
| 4146 | Seed-tree Removal Cut (w/ leave trees) |
| 4148 | Shelterwood Staged Removal Cut |
| 4151 | Single-tree selection cut |
| 4152 | Group selection cut |
| 4162 | Two-aged Coppice Cut (w/res) |
| 4175 | Two-aged Patch Clearcut (w/res) |
| 4177 | Two-aged Stand Clearcut (w/res) |
| 4183 | Two-aged Seed-tree Seed and Removal Cut (w/res) |
| 4192 | Two-aged Preparatory Cut (w/res) |
| 4193 | Two-aged Shelterwood Establishment and Removal Cut (w/ res) |
| 4194 | Two-aged Shelterwood Establishment Cut (w/res) |
| 4196 | Two-aged Shelterwood Final Removal Cut (w/res) |
| 4231* | Salvage cut (intermediate treatment, not regeneration) |
| 4232* | Sanitation Cut |

* These treatments are defined as intermediate harvests in the FS2470 silviculture practices manual. However, in the past they have sometimes been inappropriately used in place of regeneration harvests after disturbance.
